# A candidate glycoconjugate vaccine induces protective antibodies in the serum and intestinal secretions, antibody recall response and memory T cells and protects against both typhoidal and non-typhoidal *Salmonella* serovars

**DOI:** 10.3389/fimmu.2023.1304170

**Published:** 2024-01-09

**Authors:** Risha Haldar, Amlanjyoti Dhar, Debayan Ganguli, Suparna Chakraborty, Ananda Pal, George Banik, Shin-ichi Miyoshi, Santasabuj Das

**Affiliations:** ^1^ Division of Clinical Medicine, Indian Council of Medical Research (ICMR)-National Institute of Cholera and Enteric Diseases, Kolkata, India; ^2^ Division of Molecular Biology and Genomics, International Institute of Innovation and Technology (I3T), Kolkata, India; ^3^ Department of Infectious Diseases, Washington University School of Medicine at St. Louis, St. Louis, MO, United States; ^4^ BD Biosciences, Kolkata, India; ^5^ Division of Medicine, Dentistry and Pharmaceutical Sciences, Graduate School of Medicine, Dentistry and Pharmaceutical Sciences, Okayama University, Okayama, Japan; ^6^ Division of Biological Science, Indian Council of Medical Research (ICMR)-National Institute of Occupational Health, Ahmedabad, India

**Keywords:** glycoconjugate vaccine, O-specific polysaccharide (OSP), typhoidal and non-typhoidal *Salmonella* serovars, secretory IgA (sIgA), serum bactericidal assay (SBA), soft agar motility inhibition assay, antibody avidity, memory response

## Abstract

Human *Salmonella* infections pose significant public health challenges globally, primarily due to low diagnostic yield of systemic infections, emerging and expanding antibiotic resistance of both the typhoidal and non-typhoidal *Salmonella* strains and the development of asymptomatic carrier state that functions as a reservoir of infection in the community. The limited long-term efficacy of the currently licensed typhoid vaccines, especially in smaller children and non-availability of vaccines against other *Salmonella* serovars necessitate active research towards developing a multivalent vaccine with wider coverage of protection against pathogenic *Salmonella* serovars. We had earlier reported immunogenicity and protective efficacy of a subunit vaccine containing a recombinant outer membrane protein (T2544) of *Salmonella* Typhi in a mouse model. This was achieved through the robust induction of serum IgG, mucosal secretory IgA and *Salmonella*-specific cytotoxic T cells as well as memory B and T cell response. Here, we report the development of a glycoconjugate vaccine, containing high molecular weight complexes of *Salmonella* Typhimurium O-specific polysaccharide (OSP) and recombinant T2544 that conferred simultaneous protection against *S.* Typhi, *S.* Paratyphi, *S.* Typhimurium and cross-protection against *S.* enteritidis in mice. Our findings corroborate with the published studies that suggested the potential of *Salmonella* OSP as a vaccine antigen. The role of serum antibodies in vaccine-mediated protection is suggested by rapid seroconversion with high titers of serum IgG and IgA, persistently elevated titers after primary immunization along with a strong antibody recall response with higher avidity serum IgG against both OSP and T2544 and significantly raised SBA titers of both primary and secondary antibodies against different *Salmonella* serovars. Elevated intestinal secretory IgA and bacterial motility inhibition by the secretory antibodies supported their role as well in vaccine-induced protection. Finally, robust induction of T effector memory response indicates long term efficacy of the candidate vaccine. The above findings coupled with protection of vaccinated animals against multiple clinical isolates confirm the suitability of OSP-rT2544 as a broad-spectrum candidate subunit vaccine against human infection due to typhoidal and non-typhoidal *Salmonella* serovars.

## Introduction

Gram-negative enteric pathogen *Salmonella* is a significant contributor to infectious disease-associated morbidity and mortality of the populations around the world. Among different serovars that cause human infections; enteric fever, manifested by an acute febrile illness with mild to moderate gastrointestinal symptoms is caused by the typhoidal *Salmonella* strains, such as *S.* Typhi and *S.* Paratyphi and is more common in South-East Asia (1). *Salmonella* Typhimurium and *Salmonella* Enteritidis, in particular, are among the most common non-typhoidal *Salmonella* (NTS) strains causes gastroenteritis without spread of the bacteria to the blood or visceral organs in other parts of the world, such as US, UK and Africa. However, invasive NTS (non-typhoidal *Salmonella*) disease is not uncommon, especially among immunocompromised individuals with case fatality rate reaching as high as 15% (2). On the other hand, around 20% of untreated enteric fever patients die of complications like intestinal perforation or encephalopathy (2). According to estimates from the Global Burden of Disease (GBD) 2019 report by the Institute of Health Metrics and Evaluation, typhoid, paratyphoid and invasive non-typhoidal infection were responsible for 40%, 9%, and 29% of all *Salmonella* mortality with 17%, 2% and 45% deaths, respectively in under 5 children (3). Further, gallstone disease has shown an association with *Salmonella* carriage that may lead to adenocarcinoma of the gall bladder.

Vaccination remains the most attractive and immediate solution for the prevention of transmission of human *Salmonella* infections. A live attenuated (*S.* Typhi Ty21a strain) and a subunit (Vi-polysaccharide) vaccine against *S.* Typhi are globally available in different countries. However, available vaccines are of only modest efficacy in the long run, while safety and efficacy remain major concerns for the live and Vi-based vaccines, respectively in the small children (4). Recent development of Vi-polysaccharide based glycoconjugate vaccines (Vi-tetanus toxoid, Vi-diphtheria toxoid, Vi-rEPA, Vi-CRM197 etc) has generated considerable hope, but their long-term efficacy in typhoid endemic areas need further proof (5). Moreover, protection induced by the available Vi-conjugate vaccines would still depend on systemic anti-Vi antibodies (6) due to the absence of intrinsic *Salmonella* proteins and would confer little protection against *S.* Paratyphi A and B and Vi-negative *S.* Typhi strains. However, cross-protection against *S.* Paratyphi B was reported with the live typhoid vaccine (7).

In contrast to the available typhoid vaccines, no vaccine against *S.* Paratyphi and NTS (non-typhoidal *Salmonella*) infections has been licensed so far. Phase 1 studies were conducted to investigate the effectiveness of the oral, live-attenuated *S.* Paratyphi A vaccine (CVD 1902), while preclinical studies evaluated oral vaccines against *S.* Typhimurium (CVD 1921 and CVD 1941) (8). Phase 1 trial with the live vaccine WT05 against iNTS resulted in prolonged stool shedding in volunteers, leading to its abandonment. The major challenge in the development of live-attenuated vaccines is the optimal degree of attenuation without reducing the immunogenicity. While the GMMA (Generalized Modules for Membrane Antigens) vaccines against *S.* Typhimurium and *S.* Enteritidis are safer than the live vaccines and induced *Salmonella*-specific B and T cell immunity, an optimal balance between the reactogenicity and immunogenicity in humans is yet to be established (8).

Recombinant *Salmonella* proteins like flagellin and outer membrane proteins (Omp C, F, and D) were examined in the vaccination strategy that generated *Salmonella*-specific B and T cells. However, proteins having multiple membrane-spanning domains have problems maintaining their structure, leading to the induction of antibodies with poor functions. Further, such vaccines are not always accessible to simple production methods and may require a laborious procedure associated with increased risk of contamination, particularly with lipopolysaccharide (LPS) (9). In addition, FliC was reported by other studies to cause significant toxicities in mice, such as liver injury, acute cardiac dysfunction, pro-apoptotic signaling and sepsis-like systemic inflammatory response (10–12). Few published reports suggested the conserved type 3 secretion system tip and translocator proteins of NTS (non-typhoidal *Salmonella*) and their chimera as vaccine candidates for serotype-independent protection (13). In contrast, several other protein subunit vaccines provided limited protection only against the homologous NTS (non-typhoidal *Salmonella*) serovar. Overall, subunit vaccines developed against the NTS (non-typhoidal *Salmonella*) strains to-date are at best modestly efficacious, for other (14, 15).

Bacterial membrane polysaccharides have been successfully incorporated in different candidate vaccines against different pathogenic strains (16–18). *Salmonella* O-antigen (O-specific polysaccharide or OSP) is a component of the outer membrane of Gram-negative bacteria which forms the distal portion of LPS. Clinical studies have implicated it as a target for protective immunity against non-Typhi serotypes as anti-OSP antibody was able to mediate serum bactericidal activity in healthy adults and children in the United States (19). Other studies showed that OSP-specific antibodies were found to kill *Salmonella in vivo* by lowering the bacterial loads in blood, liver, and spleen following passive immunization in mice and *in vitro* studies showed complement-mediated and phagocytosis mediated bacterial killing (20). Although *Salmonella* OSP molecules in their unconjugated state have limited immunogenicity, covalent attachment to proteins significantly enhances the immune response and allows their incorporation into OSP-based vaccines (21). Several formulations with different carrier proteins (TT, DT, CRM197, FliC), chemically conjugated to OSP showed considerable promise in mouse experiments. However, the side chains attached to the common backbone of the O-antigens from different serovars make them antigenically unique (22, 23). For example, O:2 antigen is characteristic of *S.* Paratyphi A, whereas O:4 and O:4,5 is for *S.* Typhimurium. *S.* Enteritidis and *S.* Typhi share the O:9 antigen (24). As a result, different monovalent glycoconjugate vaccine formulations were combined for wider vaccine coverage against multiple serovars. For example, *S.* Paratyphi OSP-DT + *S.* Typhi Vi-DT, *S.* Typhimurium OSP-CRM_197_ + *S.* typhi Vi- CRM_197_ were evaluated in preclinical studies (25) while others were tested in phase I (*S.* Typhimurium COPS-FliC + *S.* Enteritidis COPS-FliC + *S.* Typhi Vi-TT) or phase II (OSP-TT + Vi-TT) trials (3). Despite the potential of broader protective coverage, combined glycoconjugate vaccine has several inherent limitations. Administration of multiple conjugate vaccine formulations with the same or different carrier proteins may increase the chance of carrier-specific epitope suppression (CIES) or bystander interference, as reviewed by various authors (26–28). The mechanisms related to the CIES describe the pre-existing immunity to a Carrier protein may inhibit the hapten or saccharide specific immune response connected to the same carrier. However, monovalent combination of OSP-TT examined in phase 2 study against *Salmonella* Paratyphi A showed significantly increased antibody titer against OSP (3-4-fold rise at day 180) (29). Besides this, preparation of a multi-glycoconjugate vaccine is time-consuming and costly. This underscores the need for the development of single formulation carrying multivalency (multivalent vaccines) that could offer protection against a variety of *Salmonella* serovars, both typhoidal and nontyphoidal.

Use of novel carrier proteins could overcome the limitations mentioned above and enable further development of vaccine formulations. A single vaccine formulation of *Salmonella*-specific antigens that covers different *Salmonella* serovars would be preferred. We report here the development of a glycoconjugate candidate vaccine where OSP from *S.* Typhimurium is chemically linked to the recombinant outer membrane protein rT2544 from *S.* Typhi. Previous studies from our laboratory reported strong immunogenic potential of rT2544, generating antigen-specific, opsonic antibodies and cytotoxic T cells that led to protection from bacterial challenge in mice (30). In addition to antigenicity of rT2544 and its protective efficacy against *S.* Typhi and *S.* Paratyphi, we observed strong adjuvanticity to OSP, leading to augmented anti-OSP antibody response. Thus, this study creates an opportunity to use rT2544 as a carrier protein in the glycoconjugate platform. Given that no vaccines with combined protective efficacies against typhoidal and non-typhoidal *Salmonella* are currently under clinical trials, development of an OSP-based trivalent vaccine containing rT2544 as the carrier protein could be a key step forward toward the development of a broad-specificity as well as safe and effective glycoconjugate vaccine.

## Materials and methods

### Bacterial strains, growth conditions and plasmid

*Salmonella Typhi* Ty2 and *Salmonella* Typhimirium LT2 were generous gifts from J. Parkhill, Sanger Institute, Hinxton, UK. Clinical isolates of *Salmonella* Typhimurium and *Salmonella* Enteritidis were gifted by A. Mukhopadhyay (ICMR-NICED, Kolkata, India), while clinical isolates of *Salmonella* Typhi and *Salmonella* Paratyphi A were received from IMTECH, Chandigarh. All *Salmonella* strains were grown in Hektoen enteric agar and *Escherichia coli* BL21, a kind gift from Dr. Rupak K. Bhadra (CSIR-IICB, Kolkata, India) was cultured in Luria–Bertani agar at 37°C. Liquid cultures of the bacterial strains were grown in Luria Broth. Bacterial culture media and pET-28a plasmid were purchased from BD Difco and Addgene (USA), respectively. The oligonucleotides used in this study were synthesized from IDT.

### Cloning and expression, of recombinant T2544

*t2544* ORF with four arginine coding sequences inserted at the NH2 terminus was PCR amplified, using *Salmonella* Typhi Ty2 genomic DNA as the template and the following forward and reverse primers:

FP-5’TTCGCCATGGAACGCCGCGGGATCTATATCACCGGG-3’,

RP-5’ GCCCTCGAGTTAGCGGCGAAAGGCGTAAGTAATGCC-3’.

The PCR product was cloned into pET28a at the NCoI and XhoI restriction enzymes (New England Biolabs) sites. After clone confirmation by restriction digestion, followed by sequencing (AgriGenome, India), the recombinant plasmid was transformed into *E. coli* BL21 (DE3). Transformed bacteria were inoculated into LB (BD Difco) broth (300 ml) and incubated until the OD_600_ reached 0.5. Recombinant T2544 expression was inducted by 1mM IPTG for 4h at 37°C, followed by centrifugation at 5000 g. The induction was confirmed by SDS-PAGE, stained with Coomassie Blue.

### Extraction of rT2544 from bacterial inclusion bodies and purification by ion exchange chromatography

To isolate the inclusion bodies, induced bacterial cells were resuspended in sonication buffer (30 ml) and subjected to 5 cycles of sonication on ice, with each cycle consisting of 5 pulses of 1 sec each followed by 1-minute incubation. The power output was designed to deliver a maximum of 30 watts at a frequency of 20 kHz. The sonicated pellet was collected following centrifugation at 15000 rpm for 20 min at 4°C, and washed three times with protein extraction wash buffer (20 ml). After centrifugation, recombinant T2544 was extracted from the inclusion bodies using protein extraction buffer [10 mM Tris-HCL (pH 12.0), 5ml] and analyzed by 12% SDS-PAGE. The extracted rT2544 was purified by ion exchange chromatography using UNO sphere Q resin (Bio-rad), according to the manufacturer’s protocol. A Glass econo-column (1.0 x 10 cm, Bio-Rad) was packed 50% with the resin, followed by washing with 5 column volumes (CV) of water and equilibration with 10 CV (column volume) of equilibration buffer (1X PBS, pH 7.4). The equilibrated resin was admixed with the recombinant protein (rT2544, 5ml) and kept for binding up to 3h at room temperature. The mixture was passed through the column and after washing with 3 CV (column volume) of ion exchange wash buffer, column-bound rT2544 was eluted using ion exchange elution buffer with Nacl gradient. Briefly, 1 ml of elution buffer containing 1M NaCl was inserted to the protein bound column that already had 40% of wash buffer and 1ml of eluted volume was collected. Eluted rT2544 was quantitated by Bradford Reagent (Sigma) and protein purity was determined by 12% SDS-PAGE. Protein extraction and IEC buffer compositions are mentioned in **Supplementary Table 1**.

### Extraction and purification of OSP

Lipopolysaccharide (LPS) was purified from *S.* Typhimurium, using the hot phenol method as reported earlier (31). Briefly, *Salmonella* Typhimurium LT2 strain was cultured in Luria Broth (1L) at 37°C for 10h (OD 1.8). Formalin-inactivated cells were collected by centrifugation at 5000 g and washed twice with PBS. After resuspension in PBS (30 ml), 90% phenol (HiMedia) was used for 30 min at 68°C to extract crude LPS from the cell pellet. The suspension was centrifuged at 7,300 × g at 10°C for 1 h. The aqueous layer (60 ml) was precipitated with ethanol (final concentration 75%), and the precipitate was treated with DNAse (1 μg/mL), RNase (1 μg/mL) and Proteinase K (100 μg/mL) (all purchased from Roche), followed by dialysis overnight against PBS (pH 7.4) at 4°C. Extracted LPS was used to further extract O-specific polysaccharide (OSP). to this end, LPS was incubated with 1% acetic acid (HiMedia), pH 3.0, and 100°C for 1.5 hours, followed by ultracentrifugation at 64,000 ×g for 5h at 10°C, using a WX+ Ultra series centrifuge (Thermo Scientific). The supernatant was dialyzed for 48h against PBS (pH 7.4) and OSP was purified by size exclusion chromatography.

### Conjugation of OSP and rT2544

Conjugation was performed as described earlier (31). Briefly, purified OSP (1.2 mg/ml in PBS, pH 8.5-9.0) was activated with an equal volume of cyanogen bromide (CnBr, SRL), prepared as 1.2 mg/ml in acetonitrile (Fluka). The reaction mixture was kept for 6 min on ice and the pH was maintained with 0.1M NaOH. The activated mixture was derivatized with 0.8M ADH (Sigma), dissolved in 5M NaHCO_3_ (SRL). For this reaction, the pH was adjusted to 8-8.5 with 0.1M HCl. The reaction mixture was stirred at 4°C overnight and dialyzed against 1x PBS, pH 7.4 at the same temperature for 16 h. ADH derivatized OSP was then mixed with 3 ml of recombinant T2544 (1.25 mg/ml), followed by the addition of EDAC (0.05M) to the mixture and incubated for 4h on ice. The pH of this protein-polysaccharide mixture was maintained at 5.1 to 5.5 with 0.1 M HCl. Finally, the mixture was dialyzed against 1x PBS (pH 7.4) for 48 hours at 4°C.

### Circular dichroism

Circular dichroism was performed as described earlier (32). Briefly, 1.0 ml of rT2544 (180 µg/ml) was filtered through a 0.45 µm pore-size filter to remove suspended particles, and taken in a 0.1 mm path-length quartz cuvette. Circular dichroism (CD) spectrum of the protein sample was captured at the wavelength range of 200 to 300 nm at 25°C on the Jasco-1500 spectrophotometer. A minimum of three spectra were recorded at 1 nm steps at a speed of 50 nm per minute. Baselines were subtracted and data was recorded as ellipticity (CD [mdeg]).

### Fast-performance liquid chromatography

FPLC was performed as described earlier (33). Briefly, rT2544, OSP and OSP-rT2544 (conjugate) samples in normal saline (pH 7.4) were filtered through 0.22 μm syringe filters (HiMedia) and run in a Hiload 16/60 Sephacryl S300 size exclusion column (Cytiva Life Sciences) at a flow rate of 0.5 mL/min at 4°C using Biorad NGC chromatography system. The column was previously equilibrated with normal saline (pH 7.4). The buffer solution was degassed and filtered through 0.22 µm cellulose acetate membrane filters (Millipore). The polysaccharide and the protein were detected at λ =215 nm and 280 nm, respectively.

### Dynamic light scattering

DLS was performed as described earlier (34, 35). Briefly, 1 ml of Protein, polysaccharide and conjugate samples at a concentration of 0.8-1 mg/ml were filtered through a 0.45 µm pore-size filter to remove particles, if any prior to the measurement. Hydrodynamic sizes of the samples were calculated using ZEN 3600 Malvern Zetasizer with 5 mW HeNe laser at 25°C. The dispersed light is gathered at 173° in this system, which employs backscatter detection.

### Fourier transform infrared spectroscopy

FTIR was performed as described earlier (36). Briefly, functional groups of lyophilized samples were monitored using potassium bromide (KBr) pellet method (1:100 w/w). To create translucent 1 mm pellets, potassium bromide was combined with lyophilized samples (0.8–1.0 mg) and pressed with 7500 kg for 30 seconds. Spectra were recorded using the translucent pellet in Perkin Elmer Spectrum 100 system in the spectral region of 4000–400/cm.

### Proton NMR

^1^H NMR was performed as described earlier (34). Briefly, Lyophilized samples, dissolved in 0.5 mL of D_2_O (Sigma, 99.9%) were analyzed in a 400 MHz NMR spectrometer (JOEL 400 YH) at 25°C using NMR Pipe on Mac OS X workstation. A sodium salt, TSP-D4 (0.38%) was used as a standard. The spectrometer was arranged with a 5 mm triple-resonance z-axis gradient cryogenic probe head and four frequency channels. Initial delays in the indirect dimensions were intended to provide -180° and 90° first-order phase corrections at zero and first order, respectively. States-TPPI phase cycling was used to achieve quadrature detection in the indirect dimensions with a one-second relaxation delay.

### Western blot

Western blot was performed as described earlier (37). Briefly, OSP, rT2544 and OSP-rT2544 (8 µg each), resolved in 10% SDS-PAGE were transferred to a PVDF membrane (Millipore). After blocking with 5% BSA for 1 h at room temperature, membranes were incubated overnight at 4°C with polyclonal anti-rT2544 antibody (1:5000 dilutions), raised in-house. Membranes were washed with TBS-T [Tris Buffered Saline pH 7.5, containing 0.1% Tween-20 (v/v)] for 5 times and incubated with goat anti-mouse IgG antibody (1:10000 dilutions), conjugated to horseradish peroxidase (HRP) for 1 h at room temperature. After 3 washes with TBS-T in an orbital shaker, membranes were developed by chemiluminescent reagents(SuperSignal West Pico, Thermo Fisher) and the signals were captured in ChemiDoc ™ MP imaging system (Bio Rad).

### Animal breeding, immunization, and infection

Animal breeding and experimentation were approved by the institutional animal ethical committee (PRO/192/-June 2022-25). Female, inbred C57BL/6 and BALB/c mice (5-6 weeks old) were immunized subcutaneously with OSP (8µg), rT2544 (24 µg), or OSP-rT2544 (8µg of OSP and 24 µg of rT2544) at 2-weeks intervals for 3 times. Blood samples were taken from the immunized mice on days 0, 14, 28, 38, 110, and 120 by tail snip and incubated for 30 min at 37°C and centrifuged at 1,200 × g at 4°C for 15 min and stored at −80°C. Fecal samples were collected on days 0, 14, 28, and 38, weighed, and carefully dissolved in 100 mg/ml of PBS, containing 1% BSA (SRL), centrifuged at 15,000 × g at 4°C for 10 min, and protease inhibitor cocktails (Sigma-Aldrich) were added to the supernatants before storage at −80°C. Intestinal washes were collected after sacrifice of the C57BL/6 mice (on day 38). To this end, the small intestine was removed and washed three times with 1 ml of PBS-1% BSA (BSA, SRL). Samples were centrifuged at 10,000 × g at 4°C for 10 min, and protease inhibitor cocktails (Sigma-Aldrich) were added to the supernatants before storage at −80°C. Fourteen days after the last immunization (day 42), iron-overload BALB/c mice were infected with *Salmonella* Typhi or *Salmonella* Paratyphi A, while streptomycin pre-treated C57BL/6 mice were infected with *Salmonella* Typhimurium and *Salmonella* Enteritidis as described earlier (37–39). Briefly, BALB/c mice were treated with intraperitoneal injection of Fe3^+^ as FeCl_3_ in 10^−4^ N HCl (0.32 mg per gm of body weight) along with Desferoxamine (25 mg/Kg body weight) four hours prior to the bacterial challenge. Mice were orally infected with 5 x 10^7^ CFU of *S.* Typhi or 5 x 10^5^ CFU of *S.* Paratyphi A and monitored for 10 days. C57BL/6 mice were treated with streptomycin (20 mg/mouse) orally as a beverage for 24 h. At 20 h after streptomycin treatment, water and food were withdrawn for 4 h before the mice were infected orally with 5 x 10^6^ CFU of *S.* Typhimurium or *S.* Enteritidis and monitored for 30 days.

### Enzyme-linked immunosorbent assay

ELISA was performed as described earlier (37, 40). Microtiter plates were coated with 5 µg/ml of OSP or 2 µg/ml of rT2544 and incubated at 4°C overnight. After rinsing with PBS-T (Phosphate buffer saline, containing 0.05% Tween 20), the wells were blocked using PBS, containing 1% BSA (SRL) for 1 hour at room temperature. Following further washes, plates were incubated with serum, feces, or intestinal lavage samples, diluted serially (1:200 to 1: 102400 for IgG and 1:20 to 1:20480 for IgA) for 2 hours at room temperature. Subsequently, Goat anti-mouse IgG (1:10000) or IgA (1:5000) antibodies conjugated to HRP were added to the wells and incubated for 1 h at room temperature. The immune complex was developed using tetramethyl benzidine (TMB) substrate (BD OptEIA™) and OD_450_ of the mixture was measured in a spectrophotometer (Shimadzu, Japan).

Avidity test was performed as described earlier (41). Briefly, after overnight incubation with the respective antigens (OSP, rT2544), microtiter plates were incubated with OSP-rT2544 anti-sera with a dilution of 1:100 in PBS-T. To test for the avidity of serum IgG antibody, respective antigen-antibody complexes in the wells were washed (3 times) with PBS-T, containing 6M urea before the addition of HRP-conjugated anti-mouse IgG. The avidity index was calculated by multiplying the ratio of the absorbances of the wells that were washed with and without 6M urea-containing buffer by 100.

Serum cytokines (IL-4, IL-6, IFN- γ, IL-10 and TNF-α) (Invitrogen, USA) as well as IFN-γ (Krishgen biosystem) in the co-culture supernatants of T cells and BMDCs were measured using the commercial ELISA kits following the manufacturer’s protocol.

### Serum bactericidal assay

Serum bactericidal assay was performed according to an earlier described method (38, 42). Sera collected from the immunized C57BL/6 and BALB/c mice on day 38 of first immunization were heat-inactivated at 56°C for 20 min and serially diluted in PBS (1:100 to 1:102400). A mixture comprising of 25µl of guinea pig complement (25% final concentration) and 15 µl of PBS, 50 µl of diluted heat-inactivated serum and 10 µl of diluted bacteria (310 CFU, T_0_) was prepared. The mixture was incubated for three hours (T_180_) at 37°C with gentle agitation (130 rpm). The mixtures were plated on LB agar and the plates were incubated overnight at 37°C. Bactericidal titer of the complement-containing antisera was expressed as the dilution of the serum required for the reduction of bacterial growth by 50% at T_180_ compared with T_0_. Data was analyzed using GraphPad Prism 8.0.1.244 software.

### Soft agar motility inhibition assay

A motility assay was performed as described earlier (43). Briefly, soft agar (LB medium with 0.4% Bacto agar, BD Difco) was mixed with 5% intestinal lavage from the experimental mice (collected on day 38 after the first immunization dose) and left at room temperature to dry. Bacteria (1 × 10^6^ CFU) were plated on top of the dried plates, which were incubated for 10 hours at 37°C. Bacterial motility was measured by the diameter (mm) of the clearing zones and the data was analyzed using GraphPad Prism 8.0.1.244 software.

### Memory T cell assay

Myeloid precursor cells from mouse bone marrow (BM) were used to generate dendritic cells as described earlier (30). Briefly, bone marrow cells from the femur and tibia of naïve C57BL/6 mice were cultured in RPMI 1640 medium, supplemented with murine Granulocyte-Macrophage Colony Stimulating Factor (mGM-CSF, 20 ng/mL) for 7 days. On day 7, cells were harvested and starved for 12 h in RPMI 1640 containing 1% FBS, followed by stimulation with OSP or OSP-rT2544 for 24h. CD4^+^ T cells were isolated from the spleens of the immunized mice on day 120 of the first immunization using magnetic beads (BD IMag™ anti-mouse CD4 Magnetic Particles, USA). CD4^+^ T Cells were co-cultured for 24h at 37°C in presence of 5% CO2 with antigen-pulsed BMDCs at 1:1 ratio. IFN-γ concentration was estimated in the culture supernatants by ELISA (Krishgen biosystem), while theCD4^+^ T cells were analyzed by flow cytometry for T-effector memory cell determination markers (CD4^+^CD62L^low^ CD44^hi^).

### Flow cytometry

Cells were stained with fluorochrome-conjugated anti-mouse antibodies following the standard protocol. Briefly, CD4^+^ T Cells co-cultured with antigen-pulsed BMDCs were harvested and subjected to F_c_ blocking in FACS buffer (1:50 ratio) for 20 min on ice. Following centrifugation at 1500 rpm for 5 min at 4°C, cells were stained with fluorochrome-conjugated antibodies (BD Biosciences) against specific surface markers (CD4-Percp Cy5.5, CD44- FITC, and CD62L-PE Cy7) for 30 min at 4°C in the dark. After staining, the cells were washed three times in FACS buffer and fluorescent signals were measured by FACS ARIA-II (BD Bioscience). Data were analyzed by FlowJo (version V10.8.1).

### Data analysis

Data related to CD, FPLC, DLS and FTIR were analyzed using ORIGIN software (2019b). NMR data were processed in MestReNova -9.0.1-13254 software. Antibody titers were represented as reciprocal of the log 2 values. Statistical analysis was performed using GraphPad Prism 8.0.1.244. Comparison between two groups was calculated by student t-test, while two-way ANOVA with Tukey’s *post-hoc* test was performed for multiple comparisons. Statistical significance was measured at *P <0.05, **P < 0.01, ***P < 0.001, ****P < 0.0001).

## Results

### Purification and characterization of recombinant T2544

**Figure 1:**
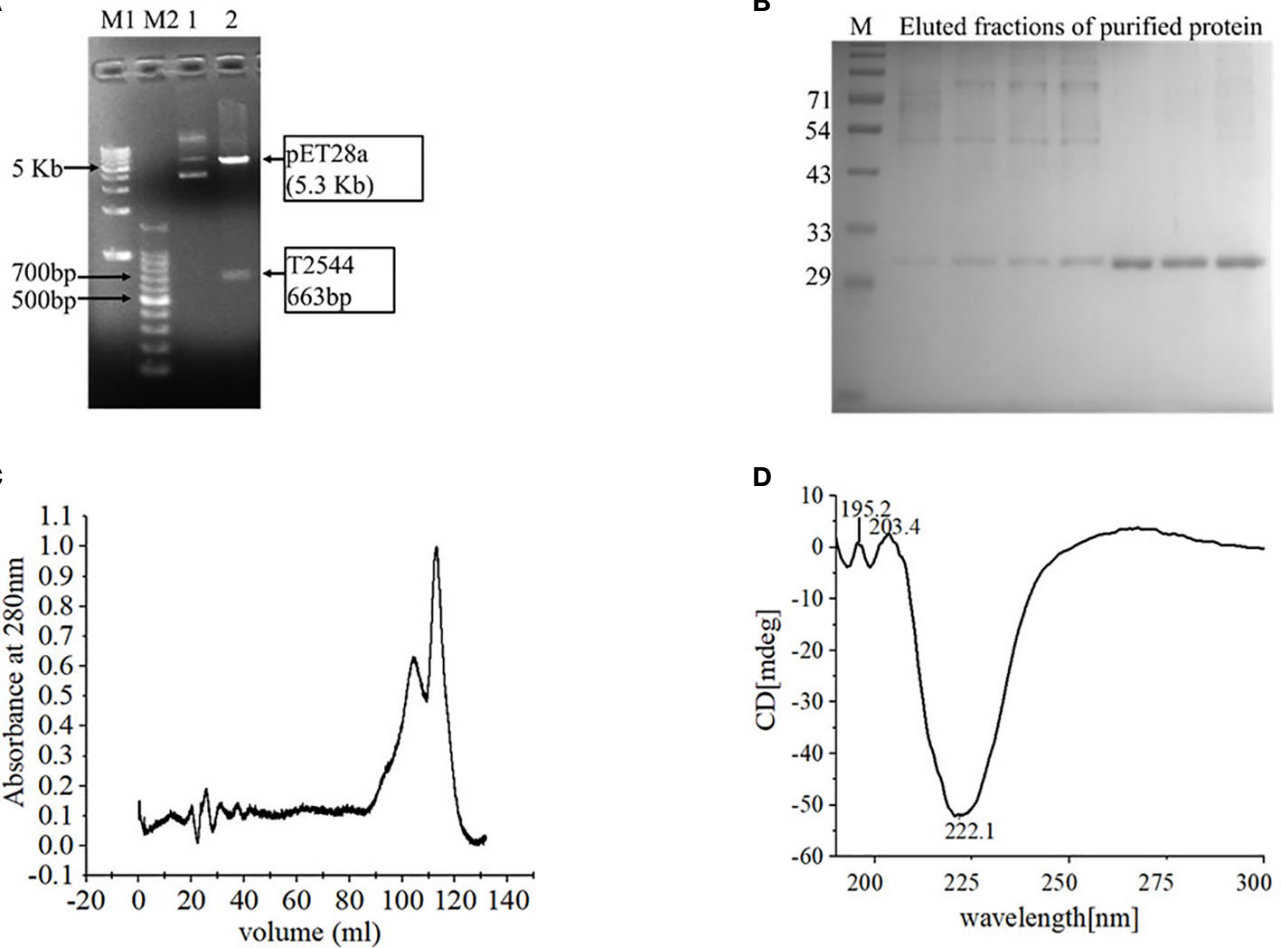
Purification and characterization of recombinant T2544. **(A)** 1% Agarose gel electrophoresis of pET28A-*t2544* after restriction digestion with XhoI and NcoI. Lane M1: 1 kb DNA ladder, M2: 100bp DNA ladder, 1: Undigested pET28A-t*2544* clone, 2: pET28A-t*2544* clone digested by XhoI and NcoI. **(B)** 12% SDS-PAGE showing the elution profile of rT2544 (3.5 µg) after ion exchange chromatography (IEC). rT2544 extracted from the inclusion bodies of E.coli was admixed with UNO sphere Q ion-exchange resin (Bio-rad), followed by binding to the Glass econo-column, 1.0 x 10 cm (Biorad) **(C)** Elution profiles of rT2544 (0.8 mg/ml) from the size exclusion chromatography column (Hiload 16/60 Sephacryl S300, Cytiva), detected at 280 nm. **(D)** Far-UV circular dichroism spectra of rT2544 (180 µg/ml) captured at the wavelength range of 200 to 300 nm at 25°C in PBS (pH 7.4) on the Jasco-1500 spectrophotometer. Data presented as ellipticity (CD [mdeg]) after subtracting the baseline values. For each analysis, experiment was replicated three times, and data from a representative experiment are shown.

Cloning of pET28a-*t2544* was confirmed by restriction digestion, followed by agarose gel electrophoresis of the digested product that showed migration of *t2455* amplicon along the predicted size of 663bp (**Figure 1A**). Nucleotide sequencing of the clone showed in-frame cloning and correct orientation of *t2544* gene (**Supplementary Figure 1A**). Recombinant T2544 (rT2544) extracted from the inclusion bodies of *E. coli* BL21 (DE3) migrated along the size of 30 kDa in SDS-PAGE (**Supplementary Figure 1B**) and ion exchange chromatography yielded a highly purified protein of the same size (**Figure 1B**). Further purification by size exclusion chromatography generated a smaller peak near 104.8 ml and a major peak near 112.8 ml fractions (**Figure 1C**). The secondary structure of rT2544 detected by Far-UV CD spectra showed one negative band at 222.1 nm, indicating alpha helix, and two positive bands at 195.2 nm and 203.4 nm, suggesting β helical structures (**Figure 1D**). Dynamic light scattering measured the hydrodynamic radius of rT2544 as 22.16 nm (**Supplementary Figure 1C**).

### Purification and characterization of OSP

**Figure 2:**
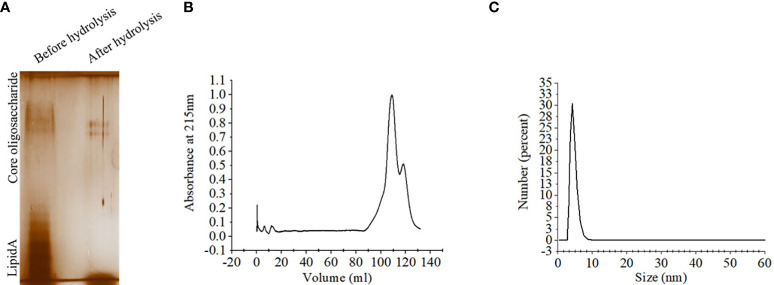
Extraction and purification of OSP. **(A)** Visualization of LPS and OSP resolved in 10% SDS-PAGE, stained by silver staining. LPS was extracted from *S.* Typhimurium LT2 strain by hot-phenol method and subjected to acid hydrolysis to isolate OSP. Lane 1: purified LPS (8 µg), Lane 2: purified OSP (8 µg). **(B)** Elution profiles of OSP (1mg/ml in normal saline, pH 7.4) eluted at a flow rate of 0.5 mL/min at 4°C from a size exclusion chromatography column (Hiload 16/60 Sephacryl S300, Cytiva), pre-equilibrated with normal saline, pH 7.4. The OSP was detected at λ =215 nm using Bio-Rad NGC chromatography system. **(C)** Dynamic light scattering (DLS) showing hydrodynamic radius (R_h_) of OSP (0.88 mg/ml, PBS, pH 7.4), determined at 25°C using ZEN 3600 Malvern Zetasizer. For each analysis, experiment was replicated three times, and data from a representative experiment are shown.

Lipopolysaccharide extracted from *S.* Typhimurium LT2 and resolved in SDS-PAGE showed multiple higher molecular weight bands and a smear corresponding to lower molecular weights upon silver staining. Acid hydrolysis of LPS removed the smear, suggesting their origin from the lipid A component, while the core oligosaccharide bands were left behind in the gel (**Figure 2A**). The gel filtration profile of OSP showed two peaks near 109.23 ml and 117.9 ml fractions that corresponded to their average K_d_ values of ~ 22.75 kDa (**Figure 2B**; **Supplementary Table 2**). ^1^H NMR analysis showed signals between 2.0 and 2.2 ppm, arising from the O-acetyl groups at C-2 of Abequose that confirmed the presence of the characteristic sugar of *S.* Typhimurium OSP. Signals between 1.79 and 1.97 ppm and 3.50 and 3.94 ppm of NMR spectra were generated from the protons bound to C-3 of Abequose and C-5 of Rhamnose and Abequose, respectively (**Supplementary Figure 2A**). Molecular radius of OSP was 4.42 nm, as calculated by DLS (**Figure 2C**). FTIR analysis disclosed the characteristic wave patterns of active OSP. Waves near 1650 cm^-1^ indicated carbonyl group (C=O), while those in the region of 1413-1261 cm^-1^ represented the deformation of C-H and C-OH groups. In contrast, waves near 1095 cm^-1^ and 1022 cm^-1^ corresponded to the characteristic peaks of the glycosidic linkage and the bands in the 936–800 cm^− 1^ region, which is called the anomeric region, indicated the α and β configuration of the anomeric carbon (**Supplementary Figure 2B**).

### OSP and rT2544 conjugation, purification and characterization of the conjugate

**Figure 3:**
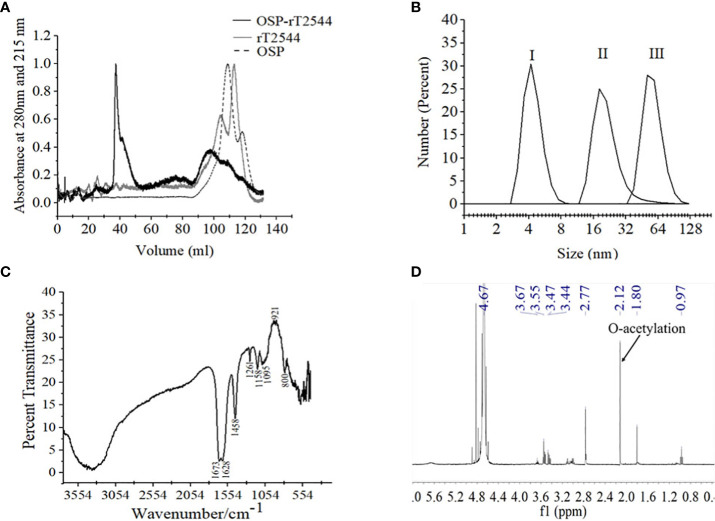
Characterization of OSP-rT2544 conjugate. **(A)** Gel filtration chromatography. OSP-rT2544 conjugate containing 1.83 mg of total protein and 0.59 mg of total sugar in normal saline (pH 7.4) was injected into Hiload 16/60 Sephacryl S300 column (Cytiva). The column was pre-equilibrated with normal saline, pH 7.4 and the flow rate was maintained at 0.5 mL/min. The conjugate (OSP-rT2544) and T2544 were observed (eluted at 41.66 ml and 112.8 ml) at 280 nm, while OSP (eluted at 109.23 ml and 117.9 ml) was observed at 215 nm at 4°C using Bio-Rad NGC chromatography system. **(B)** Dynamic light scattering (DLS) showing hydrodynamic radius (R_h_) of (I) OSP (0.88 mg/ml); (II) rT2544 (0.8 mg/ml); (III) OSP-rT2544 (1 mg/ml), dissolved in PBS (pH 7.4) at 25°C using ZEN 3600 Malvern Zetasizer. **(C)** Fourier Transform Infrared (FTIR) spectrum of the lyophilized conjugated sample (OSP-rT2544) was monitored using potassium bromide (KBr) pellet method (1:100 w/w). Spectra recorded in the Perkin Elmer Spectrum 100 system in the spectral region of 4000–400/cm indicating different functional groups of OSP-rT2544: carbonyl group (C=O) (1673 cm^-1^), C-H and C-OH groups (1458-1261 cm^-1^), glycosidic linkage (1095 and 1158 cm^-1^), aldehyde and ketone groups (921 and 800 cm^-1^) and amide bond of the protein (1628 cm^-1^). **(D)** ^1^H NMR of lyophilized OSP-rT2544, dissolved in 0.5 mL of D_2_O showing the characteristic peak of O-acetylation of OSP at C-2 of Abequose (2.12 ppm) in a 400 MHz NMR spectrometer (JOEL 400 YH) at 25°C. Additional peaks indicate protons bound to C-5 (3.4-3.6 ppm) and C-3 (1.8 ppm) of monosaccharides, the protein peaks (0.97 and 2.77 ppm) and the D2O solvent (4.67 ppm). For each analysis, experiment was replicated three times, and data from a representative experiment are shown.

Conjugates of OSP and rT2544 displayed a smear tail in the western blots, probed with T2544 antibody, indicating heterogeneity of size and mass-to-charge ratios (**Supplementary Figure 3**). FPLC analysis showed the major peak of OSP-r2544 elution after the calculated void volume (41.66 ml), as opposed to the elution peaks of OSP at 109.23 ml and 117.9 ml and rT2544 at 112.8 ml (**Figure 3A**). Higher hydrodynamic diameter of OSP-rT2544 (57.45 nm) compared with OSP (4.42 nm) and rT2544 (22.16 nm) suggested the formation of higher-order complex formation between the polysaccharide (OSP) and the protein (rT2544) (**Figure 3B**). However, we observed different molar ratios of OSP and rT2544 in the glycoconjugate molecules (**Supplementary Table 3**). To find out the functional groups in the purified OSP-rT2544 conjugate, FTIR analysis was performed. FTIR showed one additional functional group in the conjugate (OSP-rT2544), the strong amide bond of protein at 1628 cm^-1^, which was absent in OSP. On the other hand, similar functional groups in OSP-rT2544 and OSP corresponded to different wave lengths. Thus, carbonyl groups (C=O) were detected at 1673 cm-^1^, deformation of C-H, C-OH groups appeared near region 1458-1261 cm^-1^ and glycosidic linkage emerged at 1095 cm^-1^ and 1158 cm^-1^ in OSP-T2544. In addition, α and β configurations of the anomeric carbon were detected near the regions 921 cm^-1^ and 800 cm^-1^ (**Figure 3C**). However, O-acetylation pattern at C-2 of Abequose between 2.0 and 2.2 ppm was similar in OSP and OSP-T2544 in ^1^H NMR study (**Figure 3D**). But, two different peaks of the OSP-rT2544 molecule that corresponded to the aliphatic region of the protein and observed near 0.97 ppm and 2.77 ppm were absent from OSP. Total sugar and protein contents and the molecular weights of the conjugate and the un-conjugated preparations used for immunization are shown in **Supplementary Table 4**.

### Vaccination with OSP-rT2544 conferred a broad range of protection against typhoidal and non-typhoidal *Salmonella*

**Figure 4:**
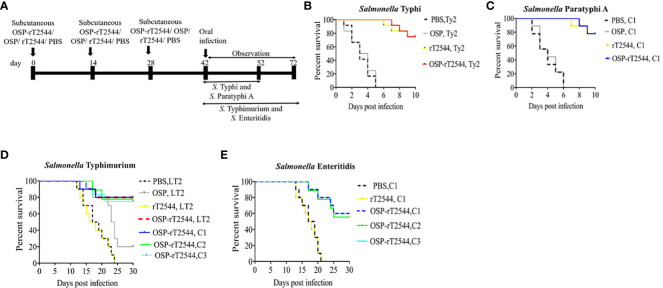
Protection of mice after subcutaneous immunization with OSP-rT2544. **(A)** Experimental scheme of mouse subcutaneous immunization with the vehicle (PBS), conjugate (OSP-rT2544) (8 µg of OSP or 24 µg of rT2544 in conjugate), or unconjugated vaccines (OSP 8 µg, rT2544 24 µg)), followed by oral bacterial challenge. **(B–E)** Kaplan-Meyer plot of cumulative mortality of the infected mice. BALB/c mice were orally challenged with *S.* Typhi Ty2 (5 x 10^7^ CFU, n= 12) **(B)** or *S.* Paratyphi A (5 x 10^5^ CFU, n=9) **(C)** and monitored for 10 days. For NTS strains, C57BL/6 mice were orally challenged with *S.* Typhimurium (5 x 10^6^ CFU of the LT2 strain (n=10), clinical strain 1 (C1, n=10), clinical strain 2 (C2, n=9) and clinical strain 3 (C3, n=12)) **(D)** or *S.* Enteritidis (5 x 10^6^ CFU of C1 (n=10), C2 (n=9) or C3 (n=10)) **(E)** and monitored for 30 days. The color scheme used to mark different experimental groups are shown in **Table 1**.

To investigate broad range efficacy of our candidate vaccine, BALB/c and C57BL/6 mice were immunized s.c. with three doses of OSP-rT2544, rT2544, OSP, or PBS (vehicle) at 14 days intervals (**Figure 4A**; **Supplementary Table 5**). Protective efficacy of the vaccines was evaluated in BALB/c mice for *S.* Typhi and *S.* Paratyphi A and in C57BL/6 mice for *S.* Typhimurium and *S.* Enteritidis strains, as mentioned under Materials and Methods (**Supplementary Table 6**). A 10xLD_50_ dose killed all the BALB/c mice within a period of 5-6 days in the vehicle- and OSP-treated groups, while 75-77% of mice that received either OSP-rT2544 or rT2544 were alive at 10 days post-infection and beyond (**Figures 4B, C**). On the other hand, OSP-rT2544 and OSP immunization protected 70-80% and 20% of C57BL/6 mice, respectively against *S.* Typhimurium infection, while all the mice that received rT2544 or the vehicle only died within 25 days (**Figure 4D**). Interestingly, 55-60% of latter mouse strain immunized with OSP-rT2544 also survived *S.* Enteritidis challenge (**Figure 4E**). Protection of the immunized mice was observed for both the reference as well as clinical strains, strongly suggesting the potential of OSP-rT2544 as a candidate quadrivalent vaccine for typhoidal and non-typhoidal *Salmonella* infections.

**Table 1:** Color scheme for different experimental groups.

Immunogen (no infection)	Color code
**Vehicle (PBS)**	**Black**
**OSP**	**Grey**
**rT2544**	**Yellow**
**OSP-rT2544**	**Orange**
Immunogen (with infection)	Color code
**Vehicle (PBS)**	**Black**
**OSP**	**Grey**
**rT2544**	**Yellow**
**OSP-rT2544 (infection with reference strains)**	**Orange**
**OSP-rT2544 (infection with C1)**	**Blue**
**OSP-rT2544 (infection with C2)**	**Green**
**OSP-rT2544 (infection with C3)**	**Sky**

### OSP-rT2544 induces protective humoral immune response against *S.* Typhi and *S.* Paratyphi A

**Figure 5:**
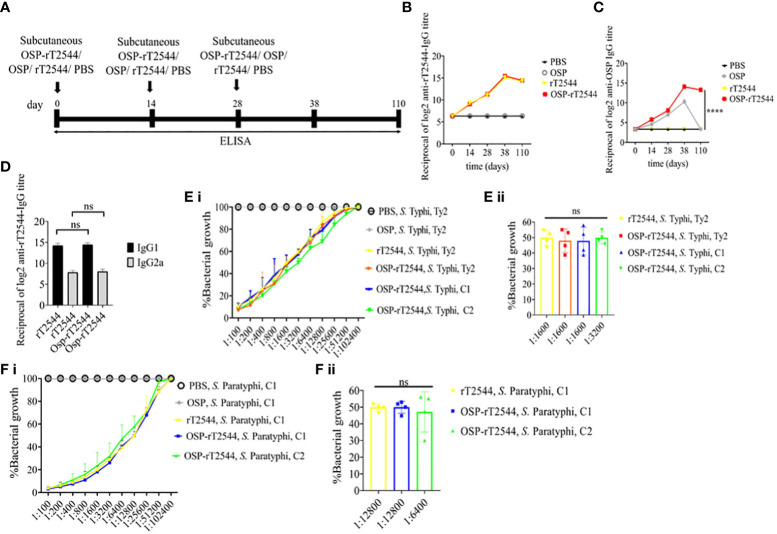
Protective antibody response against *S.* Typhi and *S.* Paratyphi A by OSP-rT2544 immunization. **(A)** Scheme of subcutaneous immunization of BALB/c mice as described under **Figure 4**. Mouse tissue samples were collected before each immunization dose and on day38 and day110 of the start of immunization. **(B, C)** Time kinetics of antigen-specific total IgG in the mouse serum as measured by ELISA. Data represent mean ± SEM values from different mice samples (n=5). X-axis indicate the time points after the start of the immunization when samples were collected. Statistical significance between the titer values from OSP-rT2544 and unconjugated OSP vaccine recipients is shown. Statistical analysis was performed using two-way ANOVA and Tukey’s post-test for multiple comparisons. *P < 0.05, **P < 0.01, ***P < 0.001. **(D)** rT2544 specific serum IgG isotypes measured by ELISA at day 38. Data represent mean ± SEM values from different mice samples (n=5). Statistical analysis was performed using two-tailed Student’s *t*-test. **(E, F)** Serum bactericidal assay. Serial dilutions of heat-inactivated antisera, collected from different groups of the immunized mice at 38 d of immunization were mixed with 25% guinea pig complement and incubated for 3h with the *S.* Typhi or *S.* Paratyphi A strains as indicated in the figure. Percentages of each live bacterial strain out of the original counts remaining at the end of the experiment for the individual serum dilutions are plotted **(E i, F i)**. Bactericidal activity was expressed as the serum dilutions at which 50% of the live bacterial loads are recovered after 3h of incubation **(E ii, F ii)**. C1, C2 are clinical isolates; bars represent the mean percent of growth reduction ± SEM of quadruplicate samples. The color scheme used are the same as above.

We had earlier reported protective humoral response in mice against *S.* Typhi upon s.c. immunization with recombinant T2544. To check if rT2544 present in the conjugate vaccine is equally immunogenic, serum antibody endpoint titers (The reciprocal of the titer (1/Y) at which the absorbance of the immune sera was the same as the control (PBS immunized sera)) were measured by ELISA, 10 days after completion of the primary immunization series as well as 110 days after the first immunization dose of BALB/c mice, immunized with OSP-rT2544 or the unconjugated vaccine formulations (**Figure 5A**; **Supplementary Table 5**). The results showed similar anti-rT2544 IgG responses at both the above time points, following vaccination with rT2544 and OSP-rT2544 (**Figure 5B**). In contrast, anti-OSP IgG titer remained significantly elevated 110 days after immunization with OSP-rT2544 only, while it touched the baseline for the mice that received unconjugated OSP, suggesting that rT2544 acted as a vaccine adjuvant to OSP (**Figure 5C**). Anti-rT2544 IgG was comprised of IgG1 and IgG2a isotypes, indicating the induction of both Th1 and Th2 type responses; however, IgG1 was the predominant isotype (**Figure 5D**). To determine the functional activities of the immune sera, bactericidal assay was performed by incubating *S*. Typhi and *S*. Paratyphi A with heat-inactivated, serially-diluted sera collected from the immunized mice, supplemented with guinea pig complement. Both OSP-rT2544 and rT2544 immune sera from BALB/c mice reduced the growth of *S.* Typhi and *S.* Paratyphi A by 50% at dilutions between 1:1600 and 1:3200 and 1:6400 and 1:12800, respectively after 3h of incubation, while unconjugated OSP immune sera displayed no growth inhibition (**Figures 5E, F**; **Supplementary Table 7**). Similar growth inhibition of *S.* Typhi and *S.* Paratyphi A was obtained for OSP-rT2544 anti-sera collected from C57BL/6 mice as well (**Supplementary Figure 4**).

### Protective humoral immune response against *S.* Typhimurium by OSP-rT2544 immunization

**Figure 6:**
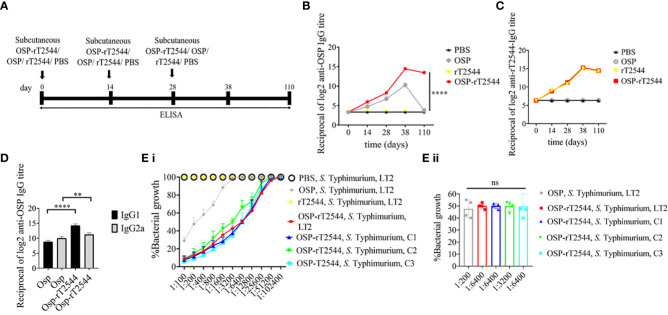
OSP-rT2544 immunization generates protective antibodies against *S.* Typhimurium. **(A)** Scheme of subcutaneous immunization of C57BL/6 mice and tissue sample collection. The scheme is similar to that described for BALB/c mice under **Figure 5**. **(B, C)** Time kinetics of antigen-specific total IgG in the mouse serum as measured by ELISA. Data presented and statistical significance calculated as under **Figure 5**. **(D)** OSP-specific serum IgG isotypes measured by ELISA at day 38. Data represent mean ± SEM values from different mice samples (n=5). Statistical analysis was performed using two-tailed Student’s *t*-test (**P < 0.01; ****P < 0.0001). **(E)** Serum bactericidal assay. SBA was performed with *S.* Typhimurium, LT2 strain or the clinical isolates, as indicated in the figure, using serial dilutions of heat-inactivated antisera and guinea pig complement as described under **Figure 5**. Bactericidal activity was expressed as above. C1, C2 and C3 are clinical isolates and bars represent the mean percent of growth reduction ± SEM of quadruplicate samples. The color scheme used are the same as above.

Given the persistently elevated, serum anti-OSP IgG titer in BALB/c mice after immunization with OSP-rT2544, we sought to investigate antisera-mediated protection against *S.* Typhimurium in similarly-immunized C57BL/6 mice by measuring antibody endpoint titers as well as SBA titers (**Figure 6A**; **Supplementary Table 5**). Like the BALB/c mice, anti-OSP IgG titer was significantly higher in OSP-rT2544 antisera than OSP antisera at day 38 of immunization and remained elevated at day 110, while OSP antisera reached the baseline (**Figure 6B**). This corroborated with correspondingly higher SBA titer of OSP-rT2544 antisera (1:6400 versus 1:200) against *S.* Typhimurium (**Figure 6E**; **Supplementary Table 7**). Similar titer values were found for OSP-rT2544 antisera from BALB/c mice to induce 50% growth reduction of *S.* Typhimurium (**Supplementary Figure 4**). As expected, there was no difference in the magnitudes of serum anti-rT2544 antibodies between the animals vaccinated with OSP-rT2544 conjugate and unconjugated rT2544 (**Figure 6C**). Markedly raised titers of OSP-specific serum IgG1 and IgG2a antibodies were observed in C57BL/6 mice immunized with OSP-rT2544, as compared with the OSP immunized mice, indicating induction of both Th1 and Th2 type responses, albeit to a significantly higher level for the later as observed for anti-rT2544 IgG isotype (**Figure 6D**). Together the above results suggested the potential for significant protection against both typhoidal and non-typhoidal Salmonellae by OSP-rT2544 antisera.

### OSP-rT2544 provides cross-protection against *S.* Enteritidis

**Figure 7:**
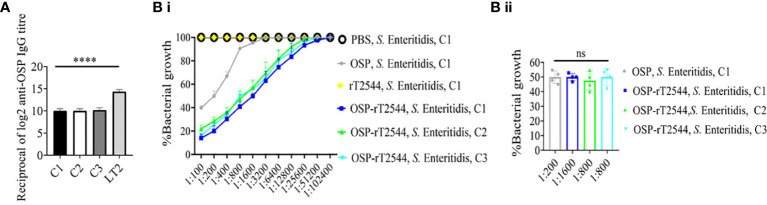
OSP-rT2544 immunization generates cross-protective antibodies against *S.* Enteritidis. **(A)** OSP was isolated from *S.* Typhimurium (LT2) and different *S.* Enteritidis (clinical isolates, C1-C3) strains and coated on the microtiter plate. Cross-reactive antibody titer was measured in OSP-rT2544 sera (38d) by ELISA. *S.* Typhimurium (LT2) OSP was used as a positive control. Data represent mean ± SEM values from different mice samples (n=6). Statistical analysis was performed using two-tailed Student’s *t*-test (****P< 0.0001). **(B)** Serum bactericidal assay. Serial dilutions of heat-inactivated antisera, collected from differentially immunized mice at 38 d of immunization were mixed with 25% guinea pig complement and incubated with the *S.* Enteritidis or clinical isolates as indicated in the figure **(B i)** Bactericidal activity was expressed as the serum dilution at which 50% growth inhibition of the bacteria was noted at T_180_ (3h incubation) compared with T_0_. Specific serum dilutions showing 50% growth reduction with individual immunogens are indicated in the figure **(B ii)**. C1, C2 and C3 are clinical isolates and bars represent the mean percent of growth reduction ± SEM of quadruplicate samples. Color scheme used is same as above.

To evaluate cross-protection against *S.* Enteritidis after immunization with OSP-rT2544 candidate vaccine, reactivity of the antisera with OSPs extracted from different *S.* Enteritidis strains was studied by measuring the titers of anti-OSP antibodies. The results showed significant cross-reactivity of OSP-rT2544 antisera with the OSPs of several clinical *S.* Enteritidis strains (**Figure 7A**). To investigate cross-protection of the antisera against *S.* Enteritidis, SBA titers were estimated as described above. The results showed 50% growth inhibition of *S.* Enteritidis by OSP-rT2544 antisera dilution of 1:800 to 1:1600 versus 1:200 dilution of OSP antisera (**Figure 7B**; **Supplementary Table 7**). Similar result was observed for the 50% growth reduction of *S.* Enteritidis when OSP-rT2544 antisera from BALB/c mice was used to perform serum bactericidal assay (**Supplementary Figure 4**). This result suggested broad range of protection against NTS strains after vaccination with OSP-rT2544 antigen.

### OSP-rT2544 candidate vaccine generates functional sIgA response, a hallmark of mucosal immunity

**Figure 8:**
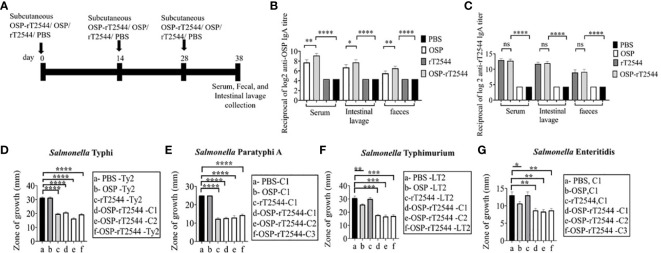
Induction of protective mucosal antibodies by OSP-rT2544 immunization. **(A)** Schedule of subcutaneous immunization of C57BL/6 mice at days 0, 14, and 28 with vehicle (PBS), conjugate (OSP-rT2544) (8µg of OSP in conjugate), or unconjugated vaccines (OSP (8 µg), rT2544 (24 µg)). Mice were sacrificed on day 38 and samples were collected. **(B, C)** ELISA showing OSP- and rT2544-specific serum IgA and intestinal sIgA titers in the groups of mice (n=5/group) after immunization with different antigens. Data represent mean ± SEM values from different mice samples (n=5). Statistical analysis was performed using two-tailed Student’s *t*-test (**P < 0.01; ***P < 0.001; ****P < 0.0001). **(D–G)** Soft agar motility assay. Bacteria were spotted at the center of the soft agar (LB medium with 0.4% Bacto agar) containing intestinal wash (5%) from the immunized mice collected on day 38. Bacterial migration from the inoculation point to the periphery of the plate was measured after 10h incubation at 37°C. Experiments were repeated three times and mean ( ± SEM) of the values from all three experiments were plotted. Statistical analysis was performed using two-tailed Student’s *t*-test (*p<0.05; **P < 0.01; ***P < 0.001; ****P < 0.0001). Color scheme used is same as above.

To study the mucosal immune response after OSP-rT2544 immunization, anti-OSP and anti-rT2544 sIgA antibodies were measured in the intestinal washes and fecal samples of the immunized mice and the titers were compared with the serum IgA titers (**Figure 8A**). Anti-OSP IgA titer was increased four times in the mice immunized with the conjugate OSP-rT2544 compared with the unconjugated OSP (**Figure 8B**). On the other hand, anti-rT2544 IgA titers were comparable for the conjugated and unconjugated vaccine recipients (**Figure 8C**). To study the functionality of intestinal secretory antibodies, inhibition of bacterial motility in soft agar motility assay by intestinal lavage from the immunized mice was performed. Motility was determined by measuring the diameter of the bacterial zones after 10 hours of incubation at 37°C (**Figures 8D–G**; **Supplementary Figure 5**). Motility inhibition of *S.* Typhi and *S.* Paratyphi A was comparable for OSP-rT2544 and rT2544 immunization. In contrast, intestinal lavage from the mice immunized with OSP-rT2544 inhibited the motility of *S.* Typhimurium significantly more (~2.5 times) than similar samples collected from OSP-immunized mice. Similar difference was observed between the two immunization groups for soft agar motility of *S.* Enteritidis (33-36% vs 15.4% inhibition). Together these results suggested that OSP-rT2544 induced functional sIgA response in the intestine against both typhoidal and non-typhoidal *Salmonella* strains.

### OSP-rT2544 induces both Th1 and Th2 serum cytokine response

**Figure 9:**
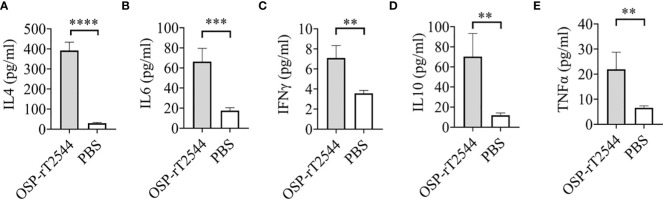
OSP-rT2544 induces both Th1 and Th2 serum cytokine response. **(A–E)** C57BL/6 mice were subcutaneously immunized with OSP-rT2544 (8µg of OSP in conjugate) or PBS (vehicle) on days 0, 14, and 28. Ten days after the last immunization (day 38), sera were collected from the immunized mice and cytokine levels in the serum were measured by ELISA. Briefly, Precoated ninety-six well plates were incubated with serum samples along with biotin-conjugate for 2h at room temperature. After three subsequent washes, plates were further incubated for one hour at room temperature with streptavidin-HRP. Following the addition of the TMB substrate, color development was evaluated using spectrophotometry at 450 nm. Statistical analysis was performed using two-tailed Student’s *t-*test (**P < 0.01; ***P < 0.001; ****P < 0.0001).

*Salmonella* clearance requires a Th1 response, whereas Th2 cells support the generation of sIgA and serum antibodies. To determine the serum cytokine response, sera were collected from the OSP-rT2544 immunized C57BL/6 mice on day 38 and cytokine concentrations were measured by ELISA. We found significantly elevated, circulating pro-inflammatory/Th1 (IFNγ, TNF-α) and anti-inflammatory/Th2 (IL-4, IL-10, IL-6) cytokines in OSP-rT2544-immunized mice as opposed to only modest elevation in the comparator immunized groups (**Figure 9**). This result suggested that OSP-rT2544 immunization induces both Th1 and Th2 cytokine response in serum with the latter being predominant.

### Immunization with OSP-rT2544 generates protective memory response

**Figure 10:**
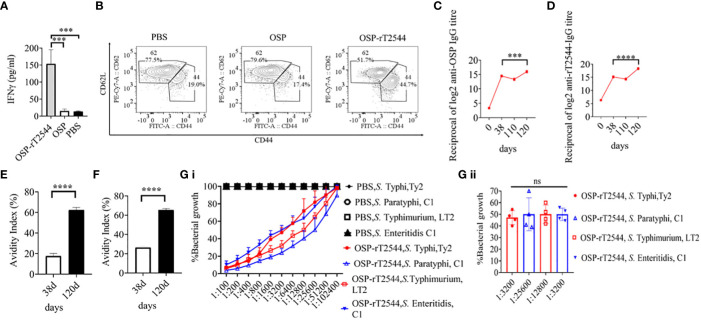
Induction of protective memory response. **(A)** C57BL/6 mice were subcutaneously immunized as described above with the antigens indicated in the **Figure 6**. Antigen-primed memory CD4+ T cells were isolated from the splenocytes of the mice 120 days of the start of the immunization. To evaluate the memory T cells response, cells were converted to effector T cells by presenting the respective antigens to them in association with MHC Class II. To this end, bone marrow derived dendritic cells (BMDCs), isolated from the naïve C57BL/6 mice were pulsed with OSP or OSP-rT2544 for 24h, followed by co-culturing of the cells with the memory T cells. Memory response was analyzed by the quantification of IFNγ released in the co-culture supernatants using ELISA. Statistical analysis was performed using two-tailed Student’s *t*-test (***P < 0.001; ****P < 0.0001). Data represents mean ( ± SEM) of four independent experiments. **(B)** CD4^+^ T cells, co-cultured with antigen-pulsed BMDCs for 24h, as mentioned under ‘Figure 10 A’, were analyzed by flow cytometry after staining for the surface expression of ‘Cluster of differentiation’ (CD) markers for T-effector memory cell determination (CD4^+^CD62L^low^ CD44^hi^). Representative images from one out of four experiments are shown. **(C, D)** C57BL/6 mice were subcutaneously immunized on days 0, 14, and 28 with OSP-rT2544 (8µg of OSP and 24 µg of rT2544 in conjugate) and a booster was given with the same antigen on day 110 **(C)** OSP and **(D)** rT2544-specific serum IgG titers were measured by ELISA at the indicated time points. Data represent mean ± SEM values from different mice samples (n=6). Statistical analysis was performed using two-tailed Student’s *t*-test (***P < 0.001; ****P < 0.0001). **(E, F)** Antibody avidity assay. Anti-rT2544 **(E)** and anti-OSP **(F)** avidity index were determined in the OSP-rT2544 serum (diluted 1:100 in PBS-T), collected at the indicated time points after washing the immune complex with 6M urea buffer by ELISA. The avidity index was calculated by multiplying the ratio of the absorbances of the wells that were washed with and without urea-containing buffer by 100. Data represent mean ± SEM values from different mice samples (n=3). Statistical analysis was performed using two-tailed Student’s *t*-test (****P < 0.0001). **(G)** Serum bactericidal assay. Serial dilutions of heat-inactivated antisera, collected from differentially immunized mice at 120 d of immunization were mixed with 25% guinea pig complement and incubated with the *S.* Typhi, *S.* Paratyphi A, *S.* Typhimurium, LT2 and *S.* Enteritidis as indicated in the figure **(G i)** Bactericidal activity was expressed as the serum dilution at which 50% growth inhibition of the bacteria was noted at T_180_ (3h incubation) compared with T_0_. Immunogens that have a 50% growth reduction at a specific serum dilution are indicated in the figure **(G ii)**. Bars represent the percent mean (± SEM) of growth reduction of quadruplicate samples. Color scheme used is same as above.

To study antigen-specific memory T cells, bone marrow derived dendritic cells (BMDCs) were isolated from the naïve C57BL/6 mice and pulsed *in vitro* with OSP or OSP-rT2544 antigen for 24h. Antigen-pulsed BMDCs were then co-cultured with the experimental mice splenocytes containing CD4^+^ T cells. IFNγ release in the co-culture supernatants was estimated to be >10 folds higher for the splenocytes from OSP-rT2544 immunized mice compared with the animals that received OSP alone or left unimmunized (13.5 pg/ml), suggesting significant augmentation of T cell memory response by rT2544 when conjugated to OSP (**Figure 10A**). To determine memory T cell subsets, co-cultured CD4^+^ T cells, as mentioned above were analyzed by flow cytometry after staining for the surface expression of ‘Cluster of differentiation’ (CD) markers (CD4^+^CD62L^low^CD44^hi^). Cell subset analysis showed that augmented memory response was largely contributed by the effector memory T cells (CD62L^low^CD44^high^) (**Figure 10B**). Further, to analyze memory B cell response, a booster dose was administered to the immunized mice on day 110 of the first immunization and anti-OSP and anti-rT2544 serum antibodies were measured ten days later. A significantly higher secondary antibody response (day 120) compared with the primary response (day 38) was observed (four times for anti-OSP, and eight times for anti-rT2544 antibodies) (**Figures 10C, D**), suggesting differentiation of memory B cells into plasma cells, producing IgG at the latter time point. Given that the avidity of antibodies for the secondary response is higher than the primary response, anti-rT2544 and anti-OSP IgG immune complexes collected at days 38 and 120 were washed (3 times) with PBS-T, containing 6M urea before the addition of HRP-conjugated secondary antibodies. The avidity index was calculated by multiplying the ratio of the absorbances of the wells that were washed with and without 6M urea-containing buffer by 100. The result showed significantly high avidity indices (60-62%) of the secondary antibodies after booster immunization compared with the primary immunization (18-22%), suggesting a strong memory B cell response (**Figures 10E, F**). To corroborate functional activities of the higher avidity antibodies, we performed serum bactericidal assay (SBA) with these antibodies and different *Salmonella* strains (**Supplementary Table 6**). The results showed 50% growth inhibition at dilutions of secondary OSP-rT2544 antisera compared with the dilutions of the primary antisera as follows, 1:3200 vs. 1:1600 for *S.* Typhi Ty2, 1:25600 vs. 1:12800 for *S.* Paratyphi A, 1:12800 vs. 1:6400 for *S.* Typhimurium LT2, 1:3200 vs. 1:1600 for *S.* Enteritidis. The result suggested that inhibition dilution of the secondary OSP-rT2544 antisera was significantly higher than inhibition accompanied by antisera collected on day 38 (**Figure 10G**; **Supplementary Table 8**). These results suggested that immunization with OSP-rT2544 might elicit potent, long-lived protective immunity against *Salmonella* infection.

## Discussion

We report here development of a glycoconjugate containing O-specific polysaccharide (OSP) from *S.* Typhimurium and an outer membrane protein (T2544) of *S.* Typhi/*S.* Paratyphi that displayed strong potential as a candidate multivalent vaccine against typhoidal and non-typhoidal *Salmonella* serovars in mouse infection models. Subcutaneous immunization of mice with OSP-rT2544 induced rapid seroconversion with high titers of protective antibodies in the serum and intestinal secretions, in addition to memory B and T cell response, conferring high protection of vaccinated animals against *Salmonella* infection.

Previous studies reported serotype independent protection against NTS (non-typhoidal *salmonella*) by S. Typhimurium type 3 secretion system tip and translocator proteins and their chimera. However, protection conferred was modest at best (13) as opposed to up to 80% protective efficacy for OSP-T2544. Bacterial surface polysaccharides are attractive candidates for vaccine development and presently constitute many commercially available vaccines. While polysaccharides, being T-independent antigens are poorly immunogenic by themselves and fail to induce immunological memory, they have been efficiently conjugated with carrier proteins to augment immunogenicity (44). Synthesis of glycoconjugate vaccines with a covalent bond between the saccharide and the carrier protein molecules and using different conjugation chemistries were described previously (45–48). The approaches taken fall into two main categories, namely the ‘random linkage’ along the polysaccharide (PS) chain and ‘selective attachment’ at the PS terminus. High molecular weight (MW), cross-linked, and generally undefined heterogeneous structures are produced by random chemistry, whereas selective chemistry generates better-defined structures while avoiding chemical alteration of the saccharide chain (45, 49–51). Immunogenicity of glycoconjugate vaccines is significantly influenced by the conjugation chemistry. Studies with OSP from different *Salmonella* strains coupled to multiple carrier proteins, using different chemical methods and diverse linkers suggested that important antigenic epitopes may be sterically protected by the bulky protein when polysaccharides are directly connected to the carrier protein (52). Instead, when a linker joins the polysaccharide to a carrier protein, steric shielding may be reduced and the polysaccharide externally presented to the immune cells, increasing the number of antigenic epitopes that are available to activate antigen-presenting cells (52). In this study, we developed the OSP-rT2544 conjugate using random linkage method where hydroxyl groups along the saccharide were randomly activated by CnBr (cyanogen bromide) chemistry (53–56). Cyanylation is a time-tested method and a simple and quick workflow for sugar-protein conjugation, as described previously for OSP-TT (31), Hib-protein conjugate (56), V. cholerae O:1 serotype Inaba (57), V. cholerae O:1 serotype Ogawa (58), and *Francisella tularensis* (59). Following cyanylation reaction, cyanate esters are formed that further interact with the hydroxyl groups to create cyclic imidocarbonates that can effectively couple to the carboxyl groups of the carrier proteins, following 1-ethyl-3-(3-dimethy laminopropyl) carbodiimide (EDC)-mediated condensation (60). For several other glycoconjugates, CDAP replaced CnBr to activate polysaccharides (31, 61). Primary mechanism of CDAP-mediated activation is the creation of isourea linkages between the cyanoesters on the activated carbohydrate and the lysine residues on the carrier protein (55, 58). However, one major drawback associated with CDAP chemistry is over-crosslinking, leading to reduced immunogenicity of the glycoconjugates due to gelling of the carbohydrates and peptides (52). In vaccine production, CnBr activation is commonly accompanied by the use of ADH linker. It was previously reported that conjugation chemistry using ADH linker is more reactive due to shorter reaction time and higher derivatization yield, as was found with the glycoconjugate vaccine for meningococcal serogroup X (62). Similar to OSP-TT (31), Vi-CRM197 (48) and Hib-protein (56) conjugates, we used ADH linker to create a covalent linkage between OSP and T2544 by carbodiimide chemistry.

Several factors, including the molecular weight (MW) of the conjugate and the molar ratio of the sugar and the carrier protein influence vaccine immunogenicity. In our study, very high molecular weight, crosslinked conjugate with partition coefficient (kd) of 0.02 was formed, as there are multiple activation points within OSP and multiple linkage points on the protein (T2544). It was previously reported that immunization with higher MW glycoconjugates results in greater anti-PS antibody response. Thus, larger and more cross-linked Vi-DT and GBS type III-TT conjugate vaccines induced higher anti-Vi and anti-saccharide IgG response, respectively (63, 64). The saccharide-to-protein ratio has a direct relationship with the immunogenicity of glycoconjugates; a larger ratio leads to improved cross-linking and activation of saccharide-specific B cells with increased polysaccharide loading.

For OSP-rT2544 conjugate containing ADH linker and generated by random activation, PS to protein molar ratio of 1.53 elicited significantly higher anti-OSP antibodies compared with OSP alone after three doses of mouse immunization. These features are in agreement with *Salmonella* Typhimurium OSP-TT conjugate with ADH linker and a saccharide to protein ratio of 0.6, produced by random activation. This conjugate was more immunogenic than the molecule generated by selective chemistry with the saccharide to protein ratio (w/w) of 0.1 (61). Further, using ADH linker rather than cystamine or SPDP increased the immunogenicity of *Salmonella* Typhi Vi conjugates, when it was coupled to recombinant Pseudomonas aeruginosa exotoxin A (rEPA) by random chemistry (65). Similar results were obtained with *S.* aureus type 8 capsular PS linked via random chemistry to rEPA or DT, where use of ADH linker yielded higher PS to protein ratio compared with cystamine or SPDP (66, 67). A study using deacylated lipopolysaccharides (LPS) from *Vibrio cholerae* O1 serotype Inaba and cholera toxin (CT) reported that random chemistry and ADH linker produced conjugates with LPS to CT ratio of 0.8 as opposed to 0.72 for single-point attachment using SPDP linker with the former being more immunogenic (68). However, *Salmonella* Enteritidis OSP directly conjugated to flagellin monomers, polymers, or CRM197 by random activation without linkers or with selective aminooxyoxime thioether chemistry using diaminooxy cysteamine and N-(γ-maleimidobutyloxy)- sulfosuccinimide ester linker induced similar IgG response and confers protection against bacterial challenge in mice (65, 69).

Carrier proteins used in the glycoconjugate preparations augment the immune response against the covalently attached polysaccharides, while the immune response specific to the protein largely remains unaltered. T2544 functions as an adjuvant to increase the serum anti-OSP antibody titer by 32 times after subcutaneous immunization of mice, keeping the levels of anti-T2544 antibodies unchanged (**Figures 5B, C**, **6B, C**). Flagellin in a conjugate formulation with *Salmonella* Enteritidis OSP enhanced anti-OSP antibody titers by 10-fold in mice after 3 intramuscular immunization doses, while anti-flagellin antibody response against the conjugate was similar to that of unconjugated flagellin (69). Similar results were observed for *Salmonella* Typhimurium when OSP was conjugated to FliC or CRM197 by random chemistry (42, 70), although anti-CRM197 antibody titer was 100-fold elevated with the conjugate. Surprisingly, much higher anti-OSP antibody response was reported here when conjugation was performed using selective chemistry. This is contrary to most other studies that reported higher antibody response with glycoconjugates developed by random chemistry rather than selective conjugation (61, 65, 68). Intraperitoneal immunization with *S.* Typhimurium OSP-porin conjugate resulted in anti-OSP and anti-porin end-points titers of 1/600 and 1/8500, respectively after 3 doses (71). This contrasts with anti-OSP and anti-T2544 end-point titers of 1/25600 and 1/51200, respectively in our study.

*Salmonella* conjugate vaccines, including the licensed products and those at the advanced stage of clinical development are largely monovalent, specifically acting against single *Salmonella* serovar. A Vi-TT conjugate vaccine has been licensed for local distribution in India (4–6). For *S.* Typhimurium, 90-100% protection was conferred by OSP-FliC, OSP-CRM197 and OSP-porin conjugates (70, 71) against clinical and reference strains of *Salmonella*. In contrast, OSP-T2544 candidate vaccine conferred 75-80% of protection against *S.* Typhi, *S.* Paratyphi, and *S.* Typhimurium and 55-60% cross-protection against *S.* Enteritidis (**Figure 4**). While the cause of cross-reactivity to S. Enteritidis is still under investigation, antibodies against common O-Ag epitopes like O:1 and O:12 or the shared core region are most likely to account for (42).

OSP- and rT2544-specific serum antibodies were comprised of both IgG1 and IgG2a sub-classes (**Figures 5D**, **6D**). Polysaccharide antigens have been found to induce IgG2 class switch in the absence of T cell engagement (72). In contrast, T cell-dependent (TD) protein antigens elicit antibodies of IgG1 subclass. Anti-polysaccharide antibodies shifted towards the IgG1 subclass in mice following conjugation to a carrier protein (73, 74). Similarly, OSP-rT2544 conjugate induced higher titers of anti-OSP IgG1 than IgG2a compared with unconjugated OSP (**Figure 6D**). This corroborates with the other published studies that reported predominantly IgG1 antibodies specific to OSP in the conjugate immunized group (42, 75). The elevated IgG1 response in conjugate compared to the unconjugated form of saccharide supports the concept that two forms of the saccharide may activate different regulating mechanisms or select B cell clones with different isotype-specificity (30). We also found increased IL4 concentrations in the conjugate antisera (**Figure 9**) that was previously reported for other glycoconjugate vaccines (29). IL-4 plays an important role in humoral immunity by inducing differentiation of Th0 into Th2 cells and mediating IgG1 antibody release, which may activate the classical complement pathway and provide long-term protection.

We measured protective efficacy of vaccine antigen-specific antibodies by serum bactericidal assay (SBA) titer and soft agar motility inhibition assay using intestinal sIgA. SBA is accepted as an *in vitro* surrogate of vaccine immunogenicity. SBA measured functional *Salmonella*-specific antibodies capable of complement-mediated bacterial killing, resulting in 50% decrease in bacterial count. OSP-rT2544 antisera displayed SBA titer of 1:1600 against *S.* Typhi and *S.* Enteritidis, while similar titers for *S.* Paratyphi A and *S.* Typhimurium attained the values of 1:12800 and 1:6400, respectively (**Figures 5E, F**, **6E**, **7B**). Published studies reported comparatively lower values of serum bactericidal titers for OSP-TT (against *S.* Typhi) and OSP-CRM197 (against *S.* Typhimurium and *S.* Enteritidis) (42, 76). We found significant inhibition of bacterial motility in soft agar by intestinal secretory antibodies, but failed to find similar studies in the published literature with other glycoconjugate vaccines. However, 3-fold increased titers of sIgA were reported after immunization with OSP-CRM197 (42) as opposed to 2-fold increase after OSP-rT2544 in the present study (**Figures 8D-G**). This might correlate with decreased motility of *Salmonella*, pre-incubated with the intestinal wash from the vaccinated mice, as was previously reported for *S.* Typhi and *S.* Paratyphi A ghost cell-based bivalent vaccine candidate (77).

In some studies, functional assays with the conjugate sera was performed by passive transfer into mice. Passive protection conferred by OSP-TT antisera administered through intraperitoneal route suggested functional serum antibodies against *S.* Typhimurium (61), where passively transferred IgM (80-100%) was more protective than IgG (20-30%). However, passive administration of rabbit antisera against OSP-porin conjugate through intravenous route showed 100% protection of mice against intraperitoneal challenge with *S.* Typhimurium (71). In other studies, opsonophagocytosis was performed to determine the functionality of the conjugate antisera. For *S.* Enteritidis COPS-FliC conjugate, pre-incubation with antisera resulted in 5% increase of opsonophagocytosis compared with the vehicle immune sera (69). We, however, did not evaluate OSP-rT2544 antisera by passive immunization or opsonophagocytosis assay.

The ability to generate robust and enduring immune memory is the hallmark of a successful vaccine and critical for the intended public health impact. An antibody recall response was demonstrated with tetrasaccharide-CRM_197_ conjugate after a booster on day 260 when the primary antibody response was reduced by two-fold compared with the titers achieved after the third dose of immunization (day 36) (78). In contrast, following vaccination with OSP-rT2544, sustained OSP- and rT2544-specific primary antibody response was observed at day 110, which further increased after the administration of a booster dose with the production of higher avidity antibodies which is a marker for T-cell dependent affinity maturation. A separate study evaluated B cell memory response by ELISPOT assay after human volunteers received a conjugate vaccine containing *Vibrio Cholerae* O1 Inaba and tetanus toxoid and reported 3.5 OSP-specific and 5 carrier protein-specific IgG spots per 10^5^ splenocytes at day 56 day (18).

To further corroborate the antibody recall response, we checked for CD4^+^ effector memory cells producing IFN-γ in the OSP-rT2544 immunized mice. Generation of recombinant T2544-specific CD4^+^ T cells was earlier reported earlier by our laboratory (30). Elevated levels of IFN-γ production was found following antigen restimulation of mouse splenocytes in the recipients of OSP-rT2544 (**Figures 10A, B**). However, similar studies were not reported earlier for glycoconjugate vaccines containing OSP.

Despite convincingly demonstrating activation of different arms of the immune system with protective serum and mucosal antibody response, conferring broad spectrum protection against typhoidal and non-typhoidal *Salmonella* serovars, this study has several limitations. We did not compare immunogenicity of candidate glycoconjugate vaccines, developed using different conjugation chemistries and having diverse linker molecules between OSP and T2544. Given that T2544 is an intrinsic *Salmonella* protein, head-to-head comparison with a different preparation, comprising of OSP linked to a non-*Salmonella* protein would provide further insights into the mechanisms underlying immune activation by glycoconjugate vaccines. Further elaboration of T cell response, including activation of different CD4^+^ cell subsets (Th9, Th17, follicular helper T cell, resident memory and central memory T cells) as well as cytotoxic T cells (central and effector memory cell) would better characterize the immune response. Finally, studies elaborating the relative contributions of different compartments of the immune system (humoral, cellular and mucosal) would help to develop newer vaccines with improved efficacies.

## Data availability statement

The original contributions presented in the study are included in the article/**Supplementary Material**. Further inquiries can be directed to the corresponding author.

## Ethics statement

The animal study was approved by ICMR-National Institute of Cholera and Enteric Diseases. The study was conducted in accordance with the local legislation and institutional requirements.

## Author contributions

RH: Conceptualization, Data curation, Formal analysis, Investigation, Methodology, Software, Visualization, Writing – original draft, Writing – review & editing. AD: Methodology, Writing – review & editing. DG: Methodology, Writing – review & editing. SC: Methodology, Writing – review & editing. AP: Methodology, Writing – review & editing. GB: Methodology, Writing – review & editing. S-iM: Funding acquisition, Writing – review & editing. SD: Conceptualization, Data curation, Formal Analysis, Funding acquisition, Investigation, Methodology, Project administration, Resources, Software, Supervision, Validation, Visualization, Writing – original draft, Writing – review & editing.
