# Supplementary material for: A candidate glycoconjugate vaccine induces protective antibodies in the serum and intestinal secretions, antibody recall response and memory T cells and protects against both typhoidal and non-typhoidal Salmonella serovars
Source: Front Immunol. 2024 Jan 9;14:1304170. doi: 10.3389/fimmu.2023.1304170 (PMC10804610; doi:10.3389/fimmu.2023.1304170)
Supplement: Supplementary file 1 [file DataSheet_1.docx]

Supplementary Material

**Supplementary Table 1. Composition of rT2544 extraction and purification buffer**

| **Protein extraction buffer from inclusion body** | **Composition** |
| --- | --- |
| Sonication buffer | 20 mM Tris-HCl, 400 mM NaCl, 1mM PMSF, lysozyme (1mg/ml), pH 8.0 |
| Wash buffer | 20 mM Tris-HCl, pH 8.0 |
| Extraction buffer | 10 mM Tris-HCl at pH 12.0 |
| **Protein purification buffer used in ion exchange chromatography** | **Composition** |
| Equilibration buffer | 10 mM Tris-HCL, pH 8.0, and 1.2 mM NaCl |
| Wash buffer | 10 mM Tris-HCL, pH 8.0, and 1.2 mM NaCl |
| Elution buffer | 10 mM Tris-HCL, pH 8.0, and 1M NaCl |

**Supplementary Table 2. K_d_ determination in OSP**

| Elution volume  (ml) | K_d_^a^ | MW(KDa)^b^ | Average MW(KDa) |
| --- | --- | --- | --- |
| 109.23 | 0.86 | 26.9 | 22.75 |
| 117.9 | 0.97 | 18.6 |  |

^a^ K_d_ values were calculated following FPLC analysis on Hiload 16/60 Sephacryl-S300 column. To determine the void volume, dextran 500KDa was used and the K_d_ values were determined using the following formulae,

K_d_= V_e_-V_0_/ V_t_-V_0_ where, V_e_ = peak elution volume (mL), V_0_= void volume (mL), V_t_=total elution volume (mL)

^b^ Molecular weight of OSP was calculated from the dextran (20, 40, 70, 500 kDa) standard curve using the formulae y = -0.68x + 1.8386.

**Supplementary Table 3. Determination of K_d_, sugar, and protein concentration in the conjugate sample**

| Sample | Elution volume (ml) | K_d_ (FPLC) | sugar (OSP)^a^  (mg/ml) | protein (rT2544)^b^  (mg/ml) | OSP to rT2544 molar ratio ^c^  (wt/wt) | MW  (KDa) |
| --- | --- | --- | --- | --- | --- | --- |
| OSP-rT2544 | 41.66 | 0.02 | 0.3 | 0.6 | 1:1.53 | 398.1 |
|  | 75.76 | 0.44 | 0.09 | 0.45 | 1:3.75 | 112.2 |
|  | 97.09 | 0.71 | 0.02 | 0.28 | 1:11.2 | 44.6 |

^a^ The concentration of the OSP was measured by phenol-sulphuric assay. Two hundred microlitre of 5% phenol was added to each tube containing 100 μl of sample (column fractions), vortexed followed by the addition of 2 ml of concentrated sulphuric acid. Reaction mixtures were cooled for 30 min, and OD was taken at 490 nm.

^b^ Protein concentration was measured by Bradford assay.

^c^ Molar ratios were calculated based on the OSP and protein molecular masses (OSP Avg. MW= 22.75 KDa, rT2544 Avg. MW= 30 KDa).

**Supplementary Table 4. Determination of total sugar, protein content, molecular weight (MW) and endotoxicity in different samples for immunization**

| Sample | Total sugar  (mg/ml) | Total protein  (mg/ml) | MW (KDa) | Endotoxicity ^a^  (EU/µg) |
| --- | --- | --- | --- | --- |
| OSP-rT2544 | 0.59 | 1.83 | 398.1 | -0.03 |
| OSP | 1.6 | - | 22.75 | -0.6 |
| rT2544 | - | 1.2 | 30 | -0.7 |

^a^ Endotoxicity was measured in the samples by LAL assay using LAL assay kit (Thermo Scientific), following the standard protocol.

**Supplementary Table 5. Vaccine formulation doses used for immunogenicity studies**

| Mice strain | Immunogen | Adjuvant | Dose of OSP  (µg/mice) | Dose of T2544  (µg/mice) |
| --- | --- | --- | --- | --- |
| C57BL/6 | OSP-rT2544  OSP  rT2544 | Alum  Alum  Alum | 8  8  - | 25.5  -  25.5 |
| BALB/c | OSP-rT2544  OSP  rT2544 | Alum  Alum  Alum | 8  8  - | 25.5  -  25.5 |

**Supplementary Table 6. Bacterial strains used for challenge study**

| Immunogen for immunization | Challenged bacterial strain | Mouse used for challenge | Mice model used for challenge study | Routes of challenge | Dose of challenge  [10X LD_50_] |
| --- | --- | --- | --- | --- | --- |
| OSP-rT2544  rT2544  PBS (Vehicle) | *S.* Typhi Ty2  *S.* Typhi clinical isolates (C1, C2, C3) | BALB/c | Iron overload model | Oral | 5 x 10^7^ CFU |
| OSP-rT2544  rT2544  PBS (Vehicle) | *S.* Paratyphi Clinical isolates (C1, C2, C3) | BALB/c | Iron overload model | Oral | 5 x 10^5^ CFU |
| OSP-rT2544  OSP  PBS (Vehicle) | *S.* Typhimurium WT (LT2)  *S.* Typhimurium clinical isolates (C1, C2, C3) | C57BL/6 | Streptomycin model | Oral | 5 x 10^6^ CFU |
| OSP-rT2544  OSP  PBS (Vehicle) | *S.* Enteritidis clinical isolates (C1, C2, C3) | C57BL/6 | Streptomycin model | Oral | 5 x 10^6^ CFU |

**Supplementary Table 7. SBA titre using 38^th^ day antisera**

| Anti-sera | SBA titre  (growth reduction up to 50%) | Bacterial strain |
| --- | --- | --- |
| **SBA for *S.* Typhi** |  |  |
| OSP-rT2544 | 1:1600 | Ty2 |
|  | 1:1600 | Clinical isolate 1 |
|  | 1:3200 | Clinical isolate 2 |
| rT2544 | 1:1600 | Ty2 |
| OSP | No 50% reduction (50%<) | Ty2 |
| **SBA for *S.* Paratyphi A** |  |  |
| OSP-rT2544 | 1:12800 | Clinical isolate 1 |
|  | 1:6400 | Clinical isolate 2 |
| rT2544 | 1:12800 | Clinical isolate 1 |
| OSP | No 50% reduction (50%<) | Clinical isolate 1 |
| **SBA for *S.* Typhimurium** |  |  |
| OSP-rT2544 | 1:6400 | LT2 |
|  | 1:6400 | Clinical isolate 1 |
|  | 1:3200 | Clinical isolate 2 |
|  | 1:6400 | Clinical isolate 3 |
| OSP | 1:200 | LT2 |
| rT2544 | No 50% reduction (50%<) | LT2 |
| **SBA for *S.* Enteritidis** |  |  |
| OSP-rT2544 | 1:1600 | Clinical isolate 1 |
|  | 1:800 | Clinical isolate 2 |
|  | 1:800 | Clinical isolate 3 |
| OSP | 1:200 | Clinical isolate 1 |
| rT2544 | No 50% reduction (50%<) | Clinical isolate 1 |

**Supplementary Table 8. SBA titre using 120^th^ day antisera**

| antisera | SBA titre  (growth reduction up to 50%) | Bacterial strain |
| --- | --- | --- |
| OSP-rT2544 | 1:3200 | *S.* Typhi, Ty2 |
|  | 1:25600 | *S.* Paratyphi Clinical isolate 1 |
|  | 1:12800 | *S.* Typhimurium LT2 |
|  | 1:3200 | *S.* Enteritidis Clinical isolate 1 |
| PBS | No 50% reduction (50%<) | *S.* Typhi, Ty2 |
|  | No 50% reduction (50%<) | *S.* Paratyphi Clinical isolate 1 |
|  | No 50% reduction (50%<) | *S.* Typhimurium LT2 |
|  | No 50% reduction (50%<) | *S.* Enteritidis Clinical isolate 1 |
