# Supplementary material for: A candidate glycoconjugate vaccine induces protective antibodies in the serum and intestinal secretions, antibody recall response and memory T cells and protects against both typhoidal and non-typhoidal Salmonella serovars
Source: Front Immunol. 2024 Jan 9;14:1304170. doi: 10.3389/fimmu.2023.1304170 (PMC10804610; doi:10.3389/fimmu.2023.1304170)
Supplement: Supplementary file 2 [file DataSheet_2.docx]

Supplementary Material

A


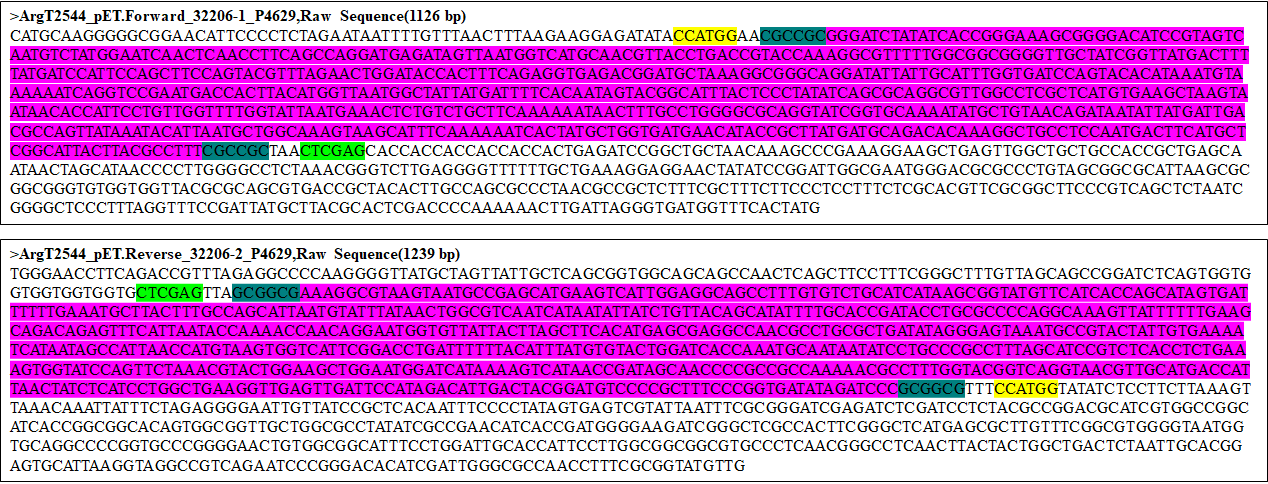


B

C


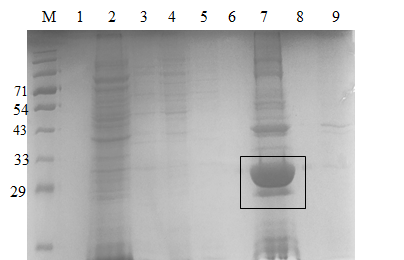




**Supplementary Figure 1. (A)** Sequencing of the pET28a-T2544 clone using pET forward and reverse primers. The FASTA file format of the sequences is provided. Yellow and green color code for the recognition sites of the restriction enzyme NcoI and XhoI, respectively, whereas magenta and blue color code for open reading frame (ORF) and arginine nucleotide sequence, respectively. **(B)** 12% SDS-PAGE of recombinant T2544 (rT2544) protein expressed in *E. coli* and extracted from inclusion bodies. After IPTG induction, inclusion bodies were sonicated, followed by successive washing and rT2544 was extracted from the inclusion bodies in protein extraction buffer, pH 12.0 (Suppl. Table 1). Lane M: Protein molecular weight marker (Prestained), Lane 1: empty lane, Lane 2: Cell lysate after sonication, Lane 3 to 4: supernatant after PBS wash, Lane 5 to 6: empty lanes, Lane 7: supernatant after re-suspension in extraction buffer, Lane 8: empty lane, Lane 9: pellet after re-suspension in extraction buffer**.** Data were replicated three times, and an image of a representative experiment is shown. **(C)** Dynamic light scattering (DLS) showing hydrodynamic radius (R_h_) of rT2544 protein (0.8 mg/ml, PBS, pH 7.4), determined at 25°C using ZEN 3600 Malvern Zetasizer. The experiment was replicated three times, and data from a representative experiment are shown.

B




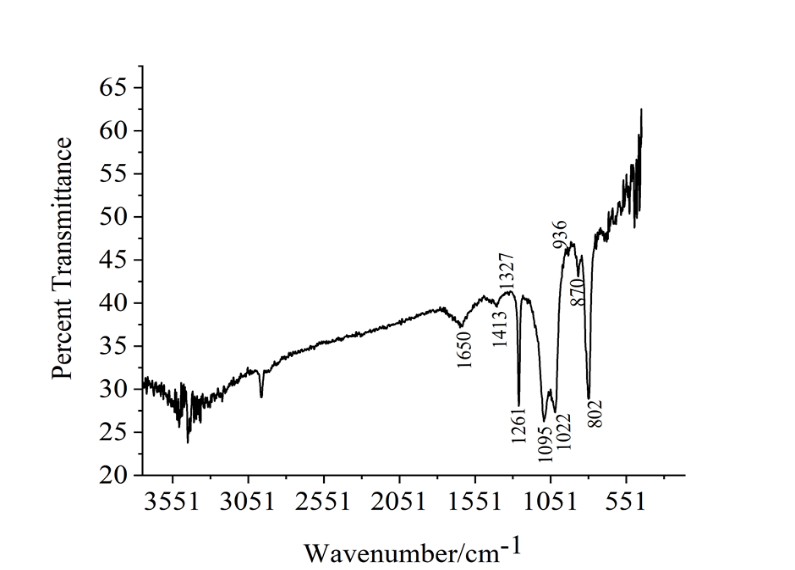


A

**Supplementary Figure 2. (A)** ^1^H NMR of lyophilized OSP, dissolved in 0.5 mL of D_2_O showing the characteristic peak of O-acetylation associated with OSP at C-2 of Abequose at 2.1 ppm. Signals between 1.79 and 1.97 ppm were generated from the protons bound to C-3 of Abequose and 3.60 and 3.94 ppm from the protons bound to C-5 of Rhamnose and Abequose in a 400 MHz NMR spectrometer (JOEL 400 YH) at 25°C. Peak arises near 4.67 ppm indicating the D_2_O solvent. **(B)** Fourier Transform Infrared spectrum (FTIR) of the lyophilized O-specific polysaccharide (OSP) was monitored using potassium bromide (KBr) pellet method (1:100 w/w). Spectra recorded in the Perkin Elmer Spectrum 100 system in the spectral region of 4000–400/ cm showing different functional groups of OSP. Waves near 1650 cm^-1^ indicated carbonyl group (C=O), while those in the region of 1413-1261cm^-1^ represented the deformation of C-H, C-OH group. In contrast, waves near 1095 cm^-1^ and 1022 cm^-1^ corresponded to the characteristic peaks of the glycosidic linkage. In addition, bands in the 936–800 cm− 1 region indicated aldehyde and ketone groups. For each analysis, experiments were replicated three times, and data from representative experiments are shown.


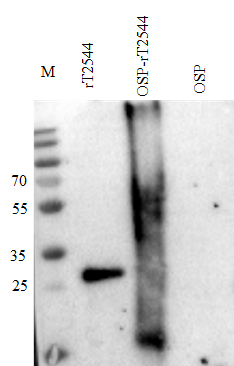


**Supplementary Figure 3.** Western blots of OSP, rT2544 and the conjugate (OSP-rT2544), resolved in 10% SDS-PAGE and probed with anti-rT2544 antibody. The experiment was repeated three times and a representative blot is shown here.

4 ii

4 i







**Supplementary Figure 4.** Serum Bactericidal assay (SBA). BALB/c (n=5) and C57BL/6 (n=5) mice were subcutaneously immunized three times with OSP-rT2544 (8 µg of OSP and 24 µg of rT2544) at days 0, 14, and 28. SBA was performed using antisera collected on day 38 after the first immunization. Serial dilutions of heat-inactivated antisera, collected from BALB/c mice were incubated with *S.* Typhimurium LT2, or S. Enteritidis C1 strain. Similarly, C57BL/6 mice sera were incubated with *S.* Typhi or *S.* Paratyphi A strains. After adding 25% guinea pig complement, bactericidal activity was expressed as the serum dilution at which 50% growth inhibition of the bacteria was noted at T_180_ (3h time point) compared with T_0_ (Figure 4 i). Specific dilutions at which 50% growth reduction for different strains was observed are indicated in Figure 4 ii. C1 is the clinical isolate and values represent mean ±SEM of four independent experiments. Statistical analysis was performed using two-tailed Student’s *t*-test.


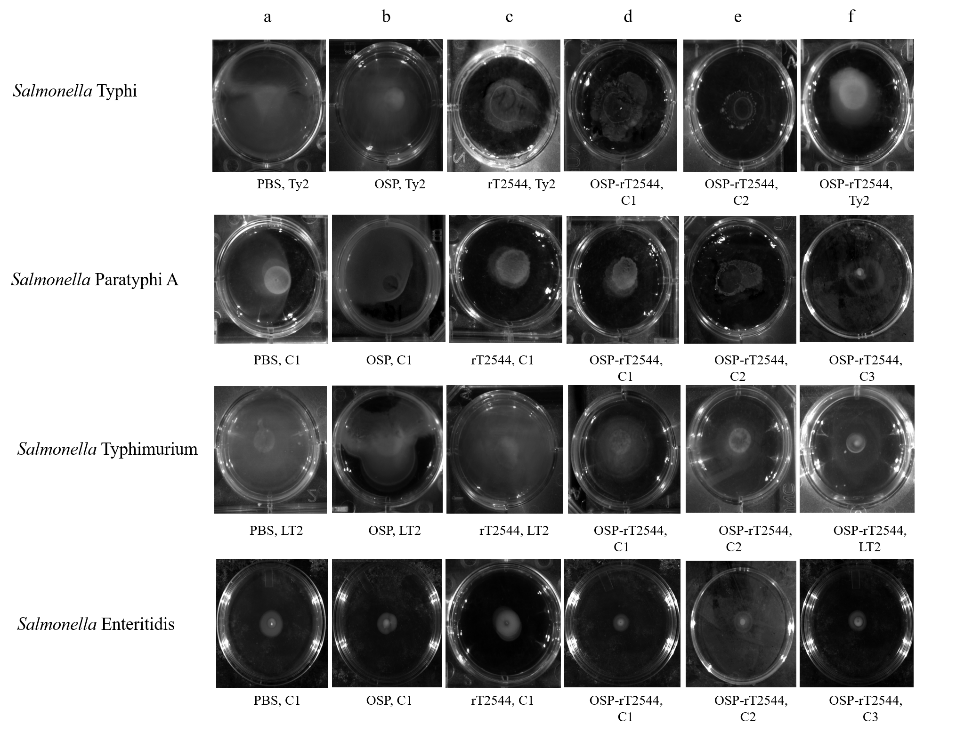


**Supplementary Figure 5.** Soft agar motility assay. The experiment was performed as described under Figure 8 D-G. Experiments were repeated three times and one representative image from three independent experiments is shown.
